# Molecular, Solid-State and Surface Structures of the Conformational Polymorphic Forms of Ritonavir in Relation to their Physicochemical Properties

**DOI:** 10.1007/s11095-021-03048-2

**Published:** 2021-05-19

**Authors:** Chang Wang, Ian Rosbottom, Thomas D. Turner, Sydney Laing, Andrew G. P. Maloney, Ahmad Y. Sheikh, Robert Docherty, Qiuxiang Yin, Kevin J. Roberts

**Affiliations:** 1 School of Chemical Engineering and Technology, State Key Laboratory of Chemical Engineering, Tianjin University, Tianjin, China; 2 Centre for the Digital Design of Drug Products, School of Chemical and Process Engineering, University of Leeds, Woodhouse Lane, Leeds, LS2 9JT UK; 3 The Cambridge Crystallographic Data Centre, 12 Union Road, Cambridge, CB2 1EZ UK; 4 Solid State Chemistry, Process R&D, AbbVie Inc, North Chicago, IL 600645 USA

**Keywords:** conformation / packing energy balance, crystal morphology, inter-molecular packing, lattice energy, molecular conformational deformation energy, particle surface energy, Ritonavir, solvent selection, surface chemistry

## Abstract

**Purpose:**

Application of multi-scale modelling workflows to characterise polymorphism in ritonavir with regard to its stability, bioavailability and processing.

**Methods:**

Molecular conformation, polarizability and stability are examined using quantum mechanics (QM). Intermolecular synthons, hydrogen bonding, crystal morphology and surface chemistry are modelled using empirical force fields.

**Results:**

The form I conformation is more stable and polarized with more efficient intermolecular packing, lower void space and higher density, however its shielded hydroxyl is only a hydrogen bond donor. In contrast, the hydroxyl in the more open but less stable and polarized form II conformation is both a donor and acceptor resulting in stronger hydrogen bonding and a more stable crystal structure but one that is less dense. Both forms have strong 1D networks of hydrogen bonds and the differences in packing energies are partially offset in form II by its conformational deformation energy difference with respect to form I. The lattice energies converge at shorter distances for form I, consistent with its preferential crystallization at high supersaturation. Both forms exhibit a needle/lath-like crystal habit with slower growing hydrophobic side and faster growing hydrophilic capping habit faces with aspect ratios increasing from polar-protic, polar-aprotic and non-polar solvents, respectively. Surface energies are higher for form II than form I and increase with solvent polarity. The higher deformation, lattice and surface energies of form II are consistent with its lower solubility and hence bioavailability.

**Conclusion:**

Inter-relationship between molecular, solid-state and surface structures of the polymorphic forms of ritonavir are quantified in relation to their physical-chemical properties.

**Supplementary Information:**

The online version contains supplementary material available at 10.1007/s11095-021-03048-2.

## Introduction

As the 20th anniversary of the seminal paper by Bauer et al (1) which described the extraordinary case of the polymorphic behavior of the active pharmaceutical ingredient (API) ritonavir has been reached, we have revisited the form I and II polymorphic structures by performing molecular, crystallographic and surface modelling calculations. These results are related to the unusual differences in physical properties between these forms, ultimately assessing how the digital workflows that are being embedded into the pharmaceutical drug R&D can unpick the complex structural chemistry that underpins polymorphic behavior of this representative API.

Ritonavir was marketed in 1996 as oral liquid solution and semi-solid capsule formulations for the treatment of Acquired Immuno-Deficiency Syndrome (AIDS) (2,3). In time, manufacturing challenges were observed when crystals of a second, previously unseen, polymorphic form appeared within the formulated drug product, resulting in a significant reduction in bioavailability and product withdrawal (1,4). The new crystals were found to be a more stable polymorphic form (form II) than that originally formulated (form I). The problem was further compounded in that the facilities which were previously making form I could now only crystallize form II, leading the compound to be labelled a ‘disappearing’ polymorph (5).

Subsequent studies revealed a total of five solid forms but, to date, only the structures of (now metastable) form I and the ‘new’ stable form II have been determined using single crystal X-ray diffraction. It was also found that the molecular conformation varied significantly between the two polymorphs and that the most stable form was also less dense than the less stable form. (1) The solubility difference between forms I and II can be up to four-fold, indicating a significant difference in solid-state energy between these two polymorphs (6).

The molecular structures in the two forms has been comprehensively evaluated, with the trans conformation of the carbamate group in form I being found to be more stable than the cis conformation in form II (7). Crystallographic analysis has shown that the hydrogen bonding pattern in form I is less optimal than in form II (8) and that solvent selection can play a role in the nucleation and polymorphic transformation process (6). Abramov et al. noted that through quantum mechanical calculations of chemical potentials, the proportions of the accessible conformations for form I and II were estimated to be 27:73% in acetone, 32:68% in propanol and 68:32% in toluene respectively (9). Despite this, there has been, as of yet, no fully detailed and integrated study that relates the molecular, solid-state and surface structure of these two forms, despite the fact that all three of these factors can play a role in the solution crystallization transition pathway from solvated molecule to crystal (9).

The recent development of digital design workflows based upon computational molecular modelling (11–15) have highlighted their potential utility in terms of assessing directed assembly and solvation (10–12); solid-state and surface properties (13–17) and formulation properties (18–20) and, through this, have provided a holistic framework for pharmaceutical product design encompassing molecular, solid-state, surface and particulate properties. This study builds upon and integrates these capabilities through a detailed examination of the structure and energetics associated with the polymorphic behavior of ritonavir forms I and II. In this, the workflow not only de-convolutes the relative energetic contributions from molecular conformational deformation and intermolecular packing (21–23) but also characterizes, at the atomic and molecular scales, the structural chemistry underpinning the distinct differences in the physical chemical properties of these two polymorphs. The latter include, most notably, their relative densities, lattice energies, surface chemistry, solubilities and crystallisabilities.

## Materials and Methods

**Fig. 1:**
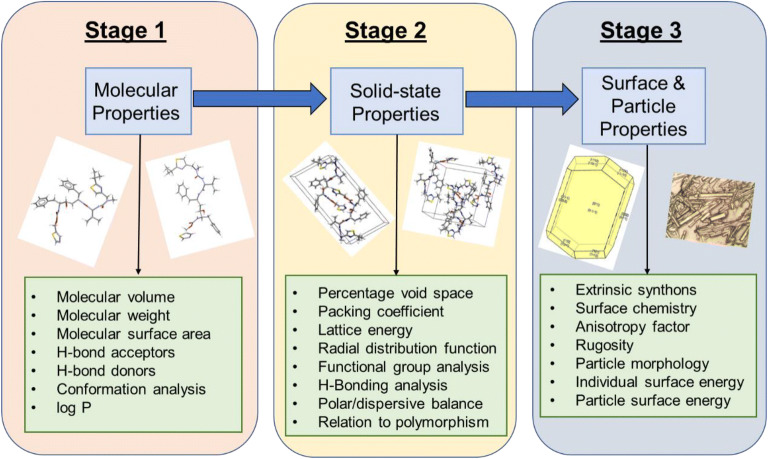
High level predictive workflow creating a digital fingerprint of the solid-state features of a new chemical entity development highlighting the 3 stage pathway from the molecular state through solid-state and surface properties to the particle properties important in formulation, overviewing the methodology for understanding the differences in structural informatics.

This paper describes the integration of a digital fingerprint of the solid state structure and particle properties into the solid form selection process using the Ritonavir case study as an example. The 3-stage computational workflow adopted in this work is summarized in Fig. 1 which highlights the inter-connectivity between the molecular, solid-state and surface and particle properties of a drug compound. This highlights how the molecular chemistry was translated through an understanding of the solid-state and surface structures to predict its anisotropic particle properties. The latter is particularly important for complex pharmaceutical molecules where the number of rotational bonds can be quite high and hence the conformational structures can be quite varied. (21)

This is useful for showing how the molecular chemistry of an organic compound is translated through its solid-state and surface structure in order to predict the anisotropic particle properties. In this, the material properties outlined are highlighted, albeit a number of other factors can also be considered, e.g. number of chiral centers, conjugated rings and ionizable groups, especially within the context of crystallizability. (24–26)

### Experimental Details

#### Materials

Crystals of ritonavir form II were supplied by Abbvie and used as provided. Acetone (HPLC grade > 99%), isopropanol (anhydrous grade > 99.5%) and toluene (reagent grade > 99.7%) solvents were supplied by VWR International, Sigma Aldrich and Sigma Aldrich, respectively, and used as supplied.

#### Preparation of Crystals for Forms I and II

Solutions of ritonavir in each of the solvents acetone, isopropanol and toluene were prepared by dissolving an appropriate amount of the as-received material in 15 g solvent, which was heated to 50°C (45°C for acetone), stirred for about 2 h and, after reaching equilibrium, filtered through a 0.45 μm filter membrane. Aliquots from the pre-prepared solutions were then transferred using pipettes to a crystallizer and maintained at the above temperature for about 25 min. Crystals of form 1 were prepared from the pre-prepared solutions, which were rapidly cooled to 10°C at 5°C/min using a Technobis (27) CRYSTAL16 crystallization system using a 1 ml sample size and a 700 rpm stirring rate which was maintained at this temperature for about 20 h. The solution supersaturations typically ranged from ca. 4.5 (acetone), 4.1 (isopropanol) and 14 (toluene). Crystals of form 2 were prepared from the pre-prepared solutions, which were transferred into 20 ml vials and cooled in a refrigerator, which was maintained at about 4°C without further stirring for more than 150 h. These conditions were consistent with a solution supersaturation of about 3. The differences in scale size used for the preparation of the two polymorphs reflected the nature of the instrumentation that was required for cooling.

Once the solutions had crystallized, the solids were isolated, dried and characterized. The melting points and enthalpy of fusion for the crystals were measured using a Mettler-Toledo DSC-1 differential scanning calorimeter over the temperature range from 140°C to 250°C using a heating rate of 25°C/min. The polymorphic form of the recrystallized materials were identified with respect to simulated powder patterns based on the single crystal structures (1) using a Bruker D8 powder X-ray diffractometer. The crystal morphology was characterized using conventional SEM and optical microscopy.

### Computational Details

#### Molecular and Crystallographic Source Structures

The molecular and crystal structures of ritonavir were extracted from the Cambridge Structural Database (28), (29) (CSD V5.39 ref. codes: form I YIGPIO02 and form II YIGPIO03), respectively). Molecular descriptors were calculated using the CSD Python script (29). Further analysis and refinement was carried out using Materials Studio V8.0 (30,31), Conquest V1.18 (32) and Mercury V3.10.3 (33). In particular, the form I structure was found to exhibit partial disorder around the isopropyl functional group and the major component of this was taken forward for the purposes of this study. Additionally, the hydrogen atom on the hydroxyl group was not provided in the data file and so this was added using the auto-hydrogen function in Materials Studio.

The resulting molecular structure for form I, together with the ‘as supplied’ structure of form II, were optimized using the Dreiding (34) forcefield in the Forcite module within Materials Studio using the Smart optimization algorithm with medium convergence criteria, whereby the molecules and conformations were allowed to relax but the unit cell was kept rigid. The intra- and inter-molecular energies were calculated using the Dreiding force-field using partial charges calculated using the Gasteiger and Marsili method, further details of the atomistic forcefield calculations are provided in supporting information S1 (35,36).

#### Molecular Conformational Analysis

The preferential geometrical orientations for the key torsions present in the molecular conformation for the two polymorphs were assessed with respect to those already present within the CSD using Conquest whilst the molecular volume, surface area and crystal packing coefficients were analyzed using Mercury (37).

The conformational energy of the isolated molecules of the two polymorphs were calculated in the gas phase by ab-initio quantum mechanical methods using Gaussian09 (38) The atomic coordinates of the molecular conformations were extracted from the optimized crystal structures of form I and II and loaded into Gaussview 5.0, without any further optimization. The single point energies of the two conformers were calculated at the density functional theory level using the 6-31G* basis set and wBD97x dispersion-corrected exchange correlation function (39).

#### Intermolecular Interaction and Lattice Energy

The intermolecular pair interaction energies together for the two polymorphic structures were calculated using HABIT98 (40–42) with Dreiding (34) force-field and MOPAC atomic charges. The energies were as partitioned into their constituent components (van der Waals, hydrogen bonded and electrostatic) and their 3D spatial arrangement within the crystal lattice was characterized using Materials Studio. The crystal lattice energy (*E*_*cr*_) due to inter-molecular packing interactions was summed as a function of radial distance. In this, the convergence was tested by increasing the intermolecular summation sphere radius of calculation to 30 Å using a step size of 1 Å with the data displayed using both cumulative and discretized radial interaction energy plots. The relative contributions of the individual atoms within the molecules were assessed through partitioning the lattice energy onto the different function groups. Through this, the structural chemistry and intermolecular energy of all the constituent inter-molecular interactions (synthons) were characterized, classified and ranked. (37,43,44)

#### Morphology Prediction and Synthon Classification

Likely morphologically-important faces associated with their growth layer thickness (d_hkl_) were identified and ranked by the BFDH method, (45–48) using Mercury (37). Dominant intermolecular interactions identified in the lattice energy calculations were partitioned between the intrinsic synthons which were fully coordinated within the growth layer (surface stability) (${E}_{sl}^{hkl}$) and the extrinsic (growth promoting) synthons (${E}_{att}^{hkl}$) associated with surface termination by the external morphology as summarized in eq. (1) thus:

1 $$ {\mathrm{E}}_{\mathrm{cr}}={\mathrm{E}}_{\mathrm{sl}}^{\mathrm{hkl}}+{\mathrm{E}}_{\mathrm{att}}^{\mathrm{hkl}} $$

The relative growth rate of each crystal habit face was taken as being proportional to ${E}_{att}^{hkl}$ (49) which was normalized with respect to the lowest growth rate and a Wulff plot (50) was used to project the predicted crystal morphology for each of the polymorphic forms. Additionally, the surface anisotropy factor (ξ_hkl_), identifying the degree of synthon saturation for the crystal surfaces (hkl), was calculated using eq. (2), thus:

2 $$ {\upxi}_{\mathrm{hkl}}=\frac{{\mathrm{E}}_{\mathrm{sl}}^{\mathrm{hkl}}}{{\mathrm{E}}_{\mathrm{cr}}} $$

#### Assessment of Surface Chemistry and Topology

The intermolecular chemistry of the selected crystal growth slices and their surface chemistry, together with their constituent synthons, were visualized using Materials Studio and tabulated on a face-specific basis.

The surface energy of the selected crystal surfaces (hkl) were calculated from the surface attachment energy (51) using eq. (3), thus:

3 $$ {\upgamma}_{\mathrm{hkl}}=\left(\frac{\mathrm{Z}{E}_{att}{d}_{hkl}}{2{\mathrm{V}}_{\mathrm{cell}}{N}_A}\right) $$

where *Z* is the number of molecules in the unit cell, *N*_*A*_ is Avogadro’s number, *V*_*cell*_ is the crystallographic unit cell volume.

The overall particle surface energy (*γ*_*particle*_) for each polymorph was estimated by calculating surface-area (SA_hkl_) weighted average of the calculated surface energies based on the predicted morphology, using eq. (4), thus:

4 $$ {\upgamma}_{\mathrm{particle}}=\sum \left(\frac{\mathrm{Z}{E}_{att}{d}_{hkl}}{2{\mathrm{V}}_{\mathrm{cell}}{N}_A}\right){M}_{hkl}\cdotp {SA}_{hkl} $$

where M_hkl_ is the multiplicity of crystal form. The fractional surface area of the habit faces (hkl) was calculated using Mercury. (37)

The inter-planar surface roughness/smoothness or rugosity of the selected crystal surfaces representing the atomic variation in height with respect to a given crystallographic plane, were calculated by taking the root mean squared variation of all the atomic positions within the asymmetric unit with respect to the surface of the crystal plane (h k l). (52)

## Results

### Molecular Properties

#### Molecular Structure and Associated Descriptors

**Fig. 2:**
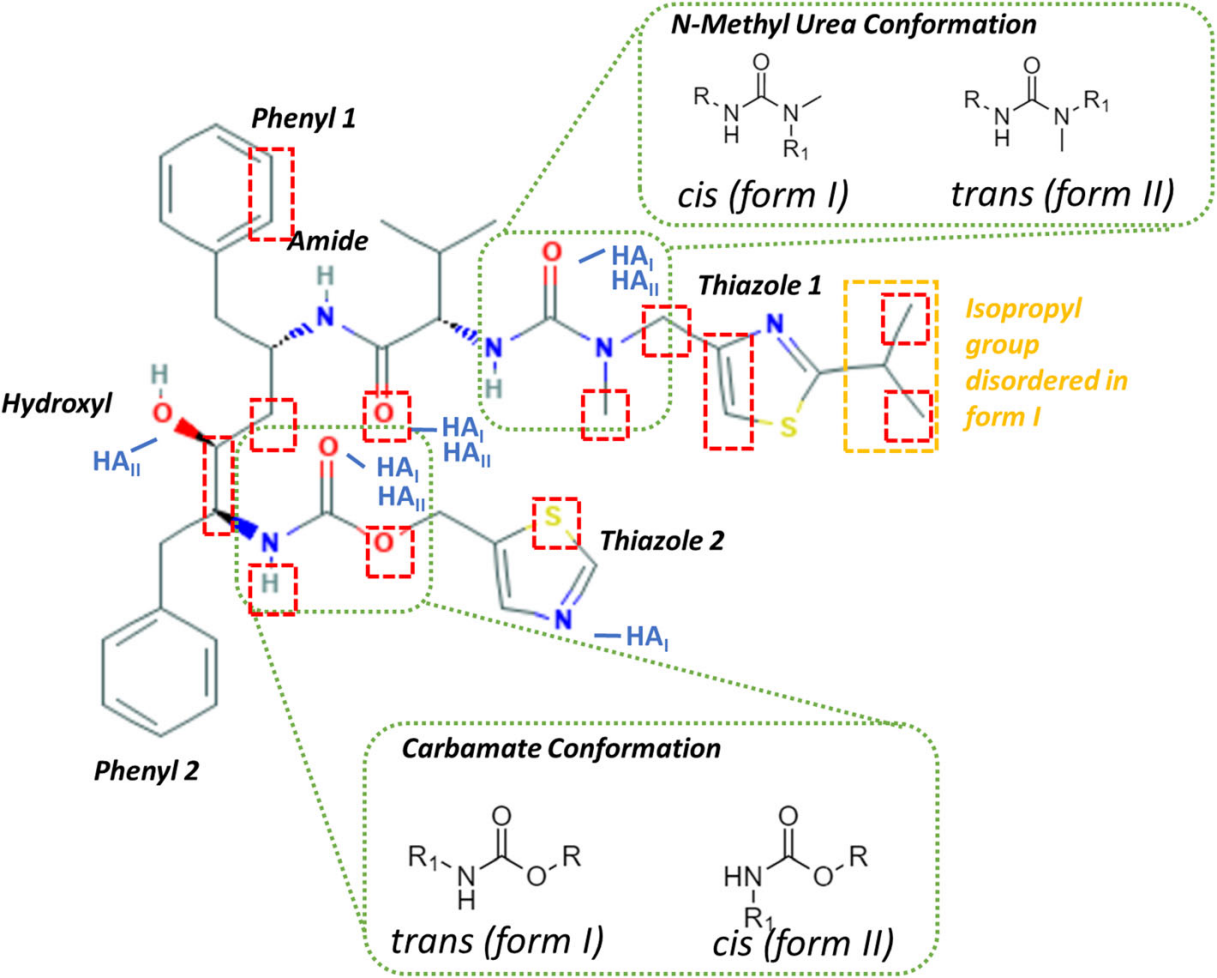
The molecular structure of ritonavir displaying the major functional groups in the molecule with the carbamate conformation highlighted in dashed green to show the trans and cis conformations of this functionality in form I and II respectively. The hydrogen bond acceptors which are active in the molecule are also highlighted with the label HA and the subscript I and II indicating the polymorphic form in which the group is active. The atoms and specific functional groups (in dashed red boxes) were found to show relatively large calculated Mopac charge differences between the form I and form II conformers. The isopropyl group at thiazole 1 (yellow dashed boxes) is disordered in the form I crystal structure.

A 2D view of the molecular structure of ritonavir is given in Fig. 2 with the molecule being separated into eight important fragments or functionalities, these include two thiazole and phenyl rings, a N-methyl urea functionality, an amide linkage, hydroxyl group and a carbamate group.

**Fig. 3:**
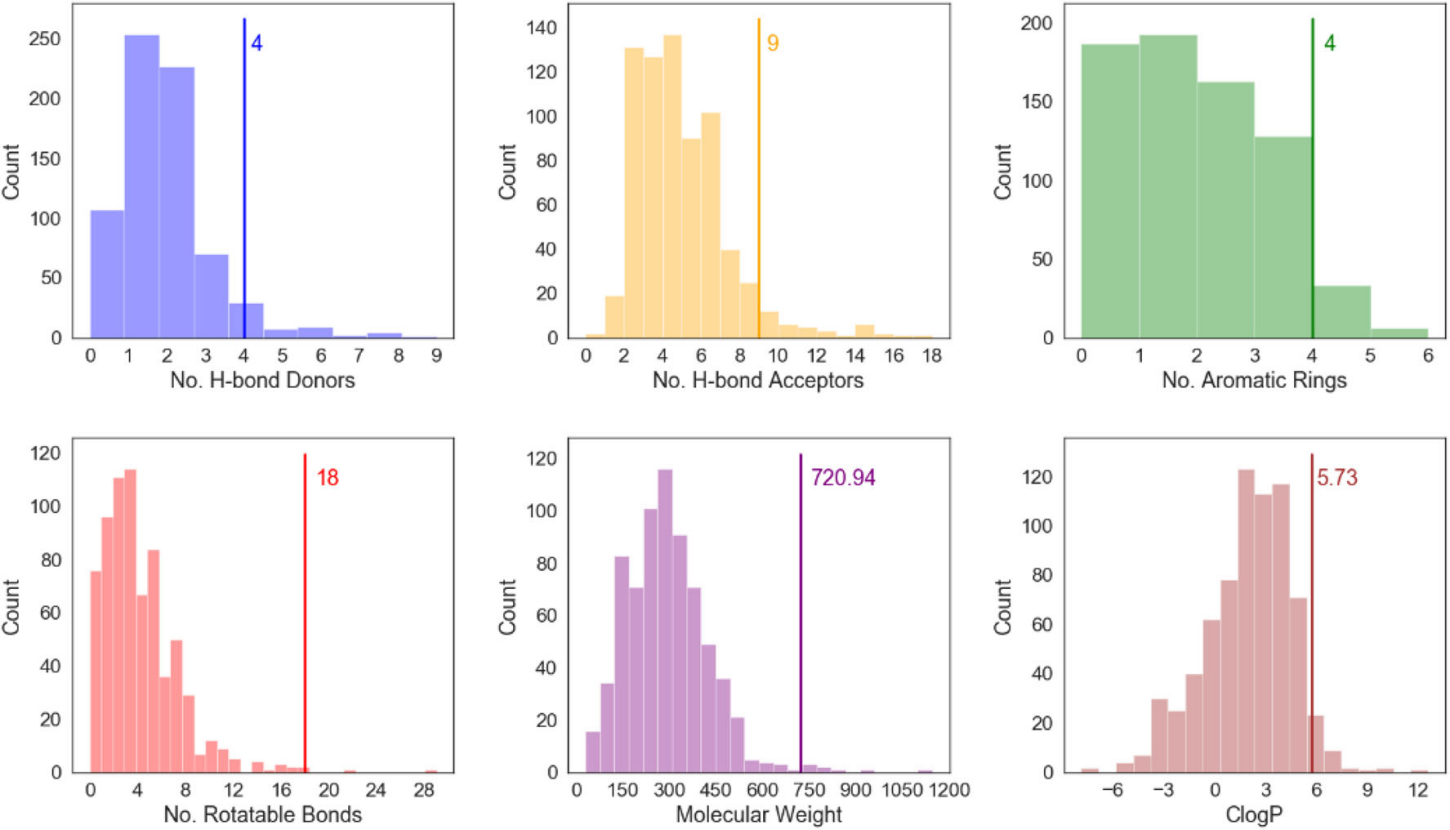
The distributions of selected molecular descriptors, relevant to drug-likeness, of single-component approved drugs in the CSD Drug Subset (59). Values of these descriptors for ritonavir are given alongside solid vertical lines. These distributions highlight ritonavir’s high molecular weight, high lipophilicity, large numbers of hydrogen bond donors and acceptors and very high number of rotatable bonds, and hence flexibility, relative to most approved drugs.

A selection of molecular descriptors for ritonavir is provided in Fig. 3 and Table II, which illustrate that ritonavir has a relatively high molecular weight (720.9 g/mol) compared to other approved pharmaceuticals (53). For a molecule of this size, it has a relatively large number hydrogen bonding options with four hydrogen bond donors, three amino protons and a hydroxyl proton, and nine potential hydrogen bond acceptors which include nitrogen and oxygen atoms of the various functionalities.

This coupled with the molecular flexibility inherent with the number of rotatable bonds (18) would be consistent with an expectation of polymorphic behavior (53). Ritonavir’s logP value of 5.73 indicates that it is highly lipophilic and this, in combination with a low aqueous solubility (54) and with a lack of any clear alignment with the Lipinski (55) criteria (detailed in S2 of supporting information) would be consistent with a class IV (56) drug compound under the biopharmaceutics classification system (BCS) (57), (58), i.e. displaying both low solubility and low permeability. The data in Table II shows that the molecular structure of the form II conformer is more compact with lower molecular volume and surface area compared to form I. This is consistent with the lower solubility observed for form II, with perhaps less opportunity for solvation.

#### Conformational Analysis and Associated Energetics

Examination of the two crystal structures reveals distinct differences in molecular conformation most notably, as highlighted in Fig. 2, associated with the carbamate and N-methyl urea group conformations which is trans and cis in form I and it is cis and trans in form II, respectively. Detailed conformational analysis (7) reveals the form I carbamate group trans conformation found to be energetically more stable than the form II cis conformation and that there is a significant energy barrier to any transformation between these two conformers.

A single point energy calculation at the DFT level estimates that the ritonavir conformer extracted from form I is ~8 kcal/mol more stable than the same extracted from form II, shown in Table I.

**Table I:** The single point energies of the ritonavir conformers extracted from the optimized crystal structures of form I and II, calculated at the wbd97xD/6-31G* DFT level of theory

Polymorphic Form	E_conf_ (kcal/mol)	ΔE_conf_ (kcal/mol)
I	−1,842,838.84	−8.09
II	−1,842,830.75	

The more stable form I conformer is closer to previous identified global minimum conformation of ritonavir (7), suggesting that it is not only more stable but is more likely to be the dominant conformer within the solution state. Minimal configurational rearrangement between solution and solid state can template the nucleation of a particular polymorph (21–23). When considering the relative stabilities of the two solid forms, such significant differences in conformational stability are likely to play a larger role than for smaller and less flexible molecules.

#### Impact of Conformational Change on Molecular Polarization

**Fig. 4:**
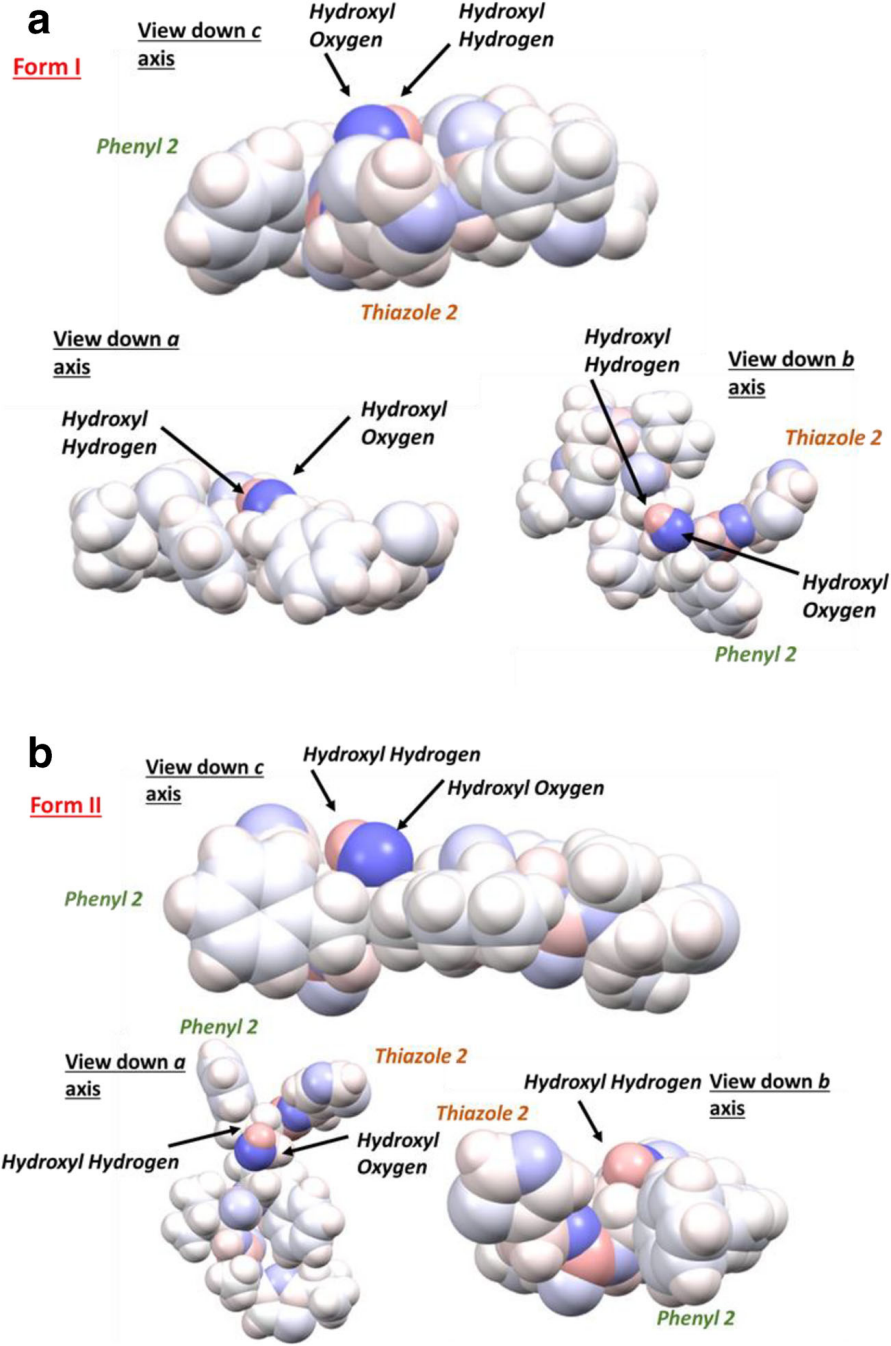
Space filling models of the molecular conformations for a) form I and b) form II where the atoms have been colored to represent their calculated charge with partial negative charge is colored blue and a partial positive charge is red. The important functional groups involved in the conformational changes between the two forms, Thiazole 2 and phenyl 2 are highlighted to show their steric position in relation to the hydroxyl group.

Examination of the calculated electronic charges distribution for the form 1 and II conformers revealed some differences between the respective molecular polarizabilities, with those atoms exhibiting the greatest differences being highlighted (in red) in Fig. 2. Analysis of this data (see full list of the calculated atomic charges, further data analysis and discussion in S3 of the supporting information) revealed that for form I, in general, the polar functional groups were found to be more polarised when compared to the form II conformer. The space filling models provided in Fig. 4 (a) and (b) provide a more detailed view of the local chemistry of the hydroxyl functionality in both forms and it can be seen that the local environment changes between the two forms due to the conformational differences between the polymorphs. Both phenyl ring 2 and thiazole 2 were found to be rotated towards the central backbone of the molecule and the oxygen of the hydroxyl group in form I. This rotation effectively creates a more compact conformation in form I as, in particular, the phenyl and thiazole rings are pushed towards the centre of the molecule and the more polar atoms. This may increase the polarization of these electronegative atoms through inductive effects. The impact of electronic partial charges on the extreme changes of solubility between similar polymeric materials has recently been shown to be a significant factor in explaining these differences rather than steric effects (60). Hence, when considering the large differences in solubility, particularly in polar solvents such as alcohol/water mixtures, the higher polarizability of the form I conformer compared to form II, could be a contributing factor for its increased experimentally determined solubility in these solvents.

#### Impact of Conformation upon Hydrogen Bonding Capacity

**Fig. 5:**
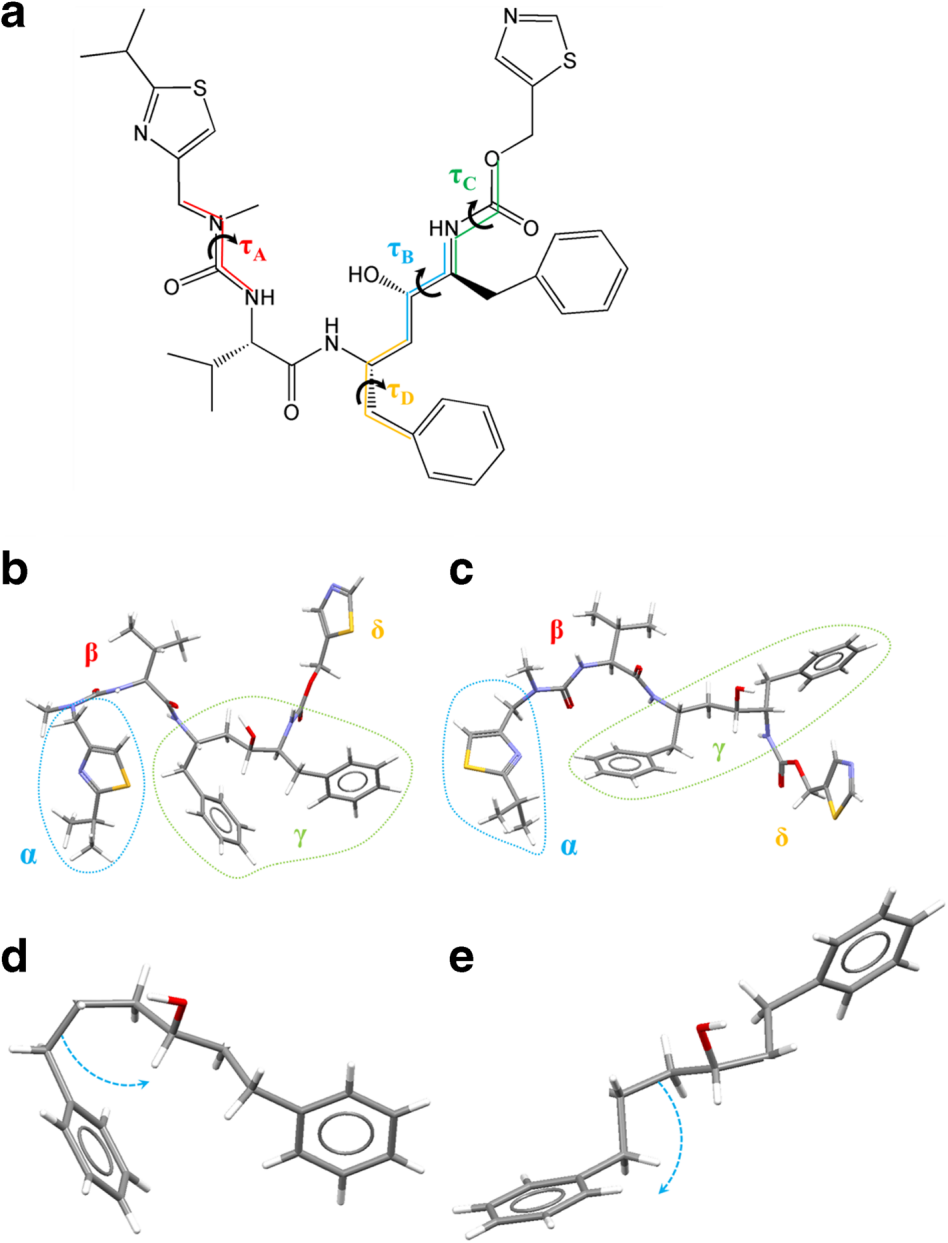
(a) Molecular structure of ritonavir; a) identification of key torsion angles; b) differences in the molecular conformation between (b) form I; (c) form II; in this, the molecular structure is segmented into four sections for ease of comparison. An enlargement of the key conformation of the γ fragment is shown in (d) for form I and (e) for form II.

A comparative examination of the two conformations is summarized in Fig. 5. Figure 5 (a) reveals the four key torsion angles (τ_A_, τ_B_, τ_C_ and τ_D_) associated with the differences between the conformations of forms I and II.

1. τ_A_ was found to involve rotation of thiazole 1 with respect to the rest of the molecule through rotation of the N-methyl urea functionality
2. τ_B_ was found to be in the center of the molecule between the carbamate and hydroxyl functionalities
3. τ_C_ was found to be the rotation of the carbamate bond involving rotation of thiazole 2 with respect to the remainder of the molecule
4. τ_D_ was found to be the rotation of phenyl 1 with respect to the rest of the molecule.

A statistical analysis of these revealed that only the τ_C_ torsion, associated with the trans conformation of the carbamate group found in form I, was found to be the dominant arrangement in the crystal structures within the CSD (1), (See further analysis and the torsion angles in supplementary material S3).

Figure 5 (b) and (c) shows how these differences impact upon the resulting rotatable molecular fragments (labelled α, β, γ and δ) notably that sections of the molecule are inversely positioned in the two forms, including the second thiazole functionality in the α group, the first thiazole functionality in the δ group and the phenyl rings in the γ group. Figure 5 (d) and (e) highlight differences in the geometry of the bulky phenyl groups around the hydroxyl group within the γ fragment, where phenyl 2 is rotated away from the hydroxyl oxygen atom in form II as previously highlighted in Fig. 4 (b). This results in a more compact conformation in form I, however the rotation of phenyl 2 away from the backbone in form II exposes the hydroxyl group, highlighted in the space filling models in Fig. 4, which is in a much better position for inter-molecular hydrogen bonding when compared to form I.

Overall, and as highlighted by previous studies, (1,7) this analysis reveals the form I conformation to be more energetically stable than that of form II, consistent with the former requiring less conformational rearrangement upon crystallization and the latter having a much slower nucleation rate (1). As shown in Fig. 3, this analysis also highlights the enhanced opportunities for intermolecular bonding afforded by the greater steric availability of both the oxygen acceptor and hydrogen donor atoms within the central hydroxyl group in the γ fragment (shown in Fig. 5 c) and d)) providing opportunities for the formation of the more complex inter-molecular interactions needed to assemble form II. This is in contrast to form I, which has a more compact conformation of the hydroxyl group where it lays comparatively flatter on the molecular surface whereas the hydroxyl group in the Form II conformation is much more open. The latter enables the hydroxyl group to be both HB donor and acceptor whereas in Form I its conformation restricts the hydrogen bonding option to being only a donor. When considered alone these conformational aspects may not seem significant. However, when integrated later with an analysis of the inter-molecular packing, mindful that the hydrogen bonding patterns of the two forms are known to be significantly different, it may be seen that the molecular conformational structural energetics and the solid-state intermolecular packing have a significant influence upon each other.

### Solid-State Properties

Available, crystallographic and thermodynamic data together with further analysis through this study is summarized in Table II.

**Table II:** Characteristic molecular descriptors and crystallographic structural data for the ritonavir polymorphs. Note the more close-packed (higher density) but larger molecular volume and surface area for form I when compared to form II

Material Descriptor	Form I	Form II
Refcode	YIGPIO02 ^(1)^	YIGPIO03 ^(1)^
Molecular volume (Å^3^)	721.24	676.74
Molecular surface area (Å^2^)	656.46	645.09
Melting point (°C)	123^(1)^	126(1)^*^
ΔH_fus_ (kcal/mol)	13.47 ^(1)^	15.12 ^(1)^
ΔS_fus_ (kcal/mol)	0.0339^(1)^	0.0380^(1)^
Space Group	P2_1_^(1)^	P2_1_2_1_2_1_^(1)^
Z / Z’	2 / 1^(1)^	4 / 1^(1)^
*a* (Å)	13.344^(1)^	9.831^(1)^
*b* (Å)	5.2150 ^(1)^	18.485^(1)^
*c* (Å)	26.693^(1)^	20.261^(1)^
α (°)	90^(1)^	90^(1)^
β (°)	103.456^(1)^	90^(1)^
γ (°)	90^(1)^	90^(1)^
Cell volume (Å^3^)	1806.55^(1)^	3681.95^(1)^
Packing coefficient	0.80 ^(this study)^	0.73^(this study)^
Void space (%)	0.3^(this study)^	0.9^(this study)^
Density (g/cc)	1.28^(1)^	1.25^(1)^

The data reveals higher melting points and enthalpy/entropy of fusions for form II consistent with this being the more stable of the two polymorphs. The lower entropy of fusion (*ΔS*_*fus*_) for form I compared to form II suggests its lower disorder increase on melting which consistent with this form I having a disordered structure (1). Whilst, form II has a lower molecular volume (see Table II), it nonetheless has a lower density and packing coefficient with a higher void space when compared to the more close-packed form I structure.

#### Lattice Energies and their Convergence

**Fig. 6:**
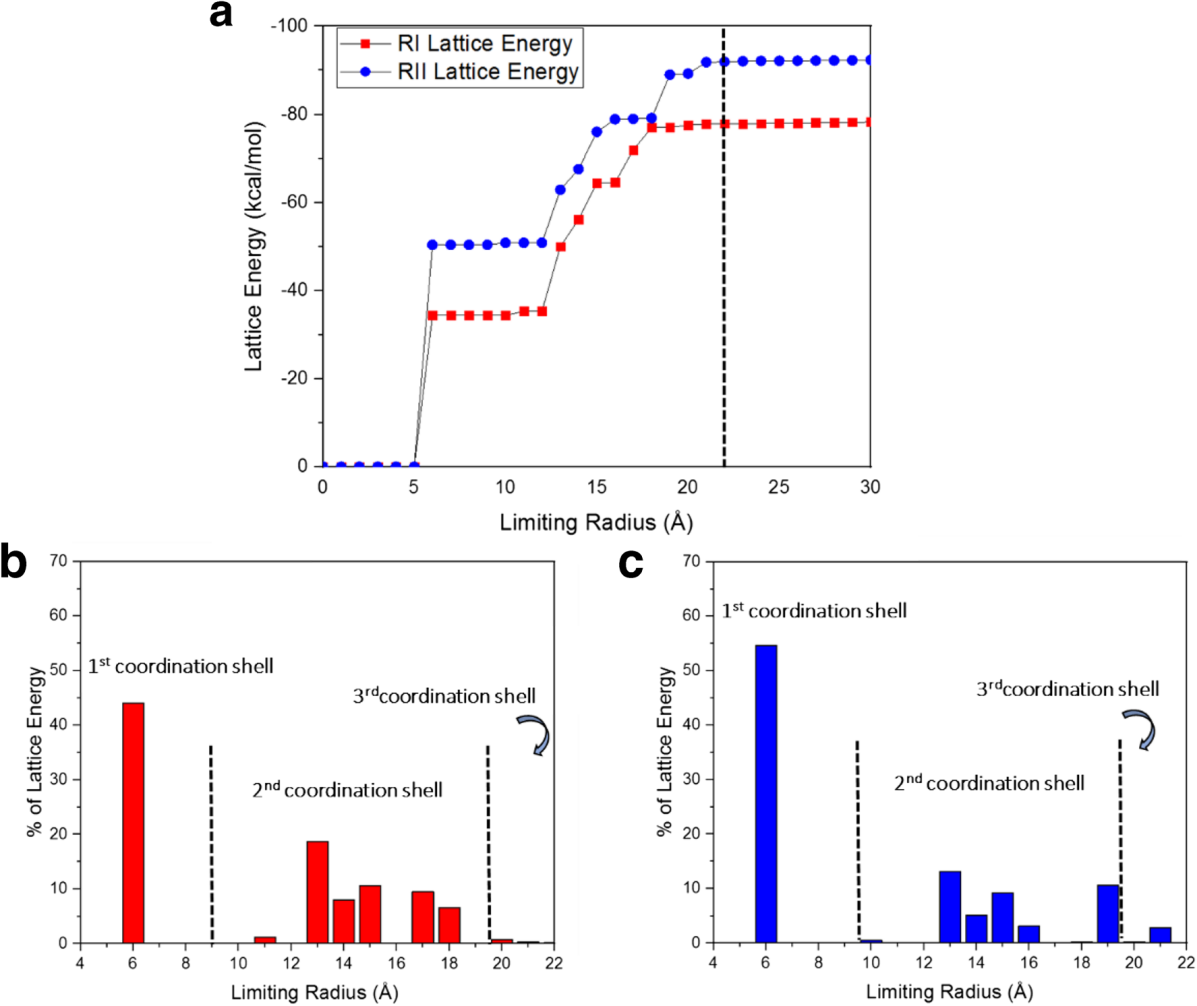
Convergence of the intermolecular summation associated with the determination of (a) the lattice energy showing the contribution of electrostatic interactions to the overall lattice energy (b, c) radial discretized distribution plots showing the % contribution to the lattice energy as a function of intermolecular summation distance for (b) form I and (c) form II.

Figure 6 shows the lattice energy convergence as a function of the limiting inter-molecular summation radius using both cumulative (a) and discretized (b) plots revealing significant differences with form II (92.30 kcal/mol) ca. 18% greater than form I (78.29 kcal/mol) This difference is significantly larger than might be expected based upon the of 1.7 kcal/mol difference between the enthalpies melting for the two polymorphs and when compared to other polymorphic materials. In the latter case the total lattice energy for ca. 95% of the polymorphs investigated were found to vary by < ca. 1.7 kcal/mol, albeit such studies have not focused upon the higher molecular weight compounds such as ritonavir (61,62). However, the calculations presented here only take into account the inter-molecular packing interactions, hence neglecting the negative contribution of 8.09 kcal/mol (Table I) due to the conformational deformation in the form II structure. When this is accounted, the lattice energy difference could be estimated to be approximately 6.00 kcal/mol i.e. ca. 7%, consistent with previously published lattice energy differences.

Despite the predicted closeness in lattice energies and small differences in enthalpy of melting previously observed, it is noteworthy that the observed solubility of form I is ca. 2.6 times greater than form II in ethanol at 25°C (1,63) increasing to 3.9–5.7 times greater in ethanol/water mixtures at 5°C. (64) The packing of form II is significantly more stable than form I, whereby it could be classified as a crystal form where the extremely stable solid-state packing limits the solubility (65). Further, one should not discount the possibility that the different molecular conformers could be wetted differently by the solvent, which might result in different solvation energies of the two forms even after dissolution. Nonetheless, this work highlights the need for further work to investigate this particular system and other more representative current pharmaceutical compounds using techniques such as e.g. statistical (66), quantum chemistry (65) and free energy perturbation models. (67)

Closer examination of the data in Fig. 6, summarized in Table III, shows that the lattice energy for form I converges at a shorter radial distance (ca. 18 Å) when compared to form II (ca. 21 Å) consistent with the formation of thermodynamically stable nucleation clusters at a size which are smaller for the metastable form I compared to stable form II (62) as might be expected given the former’s higher density (Table II). This observation agrees with known industrial crystallization data, using anti-solvent drown-out processes to produce high solution supersaturation and smaller cluster sizes (68), results in the formation of form I initially with form II only crystallizing after a lengthy induction times (1,6,64,69).

**Table III:** The percentage of the lattice energy added and the number of molecules with the increase intermolecular summation distance covering the various coordination shells

Coordination Shells	Distance Range (Å)	Number of Molecules		% Lattice Energy	
		Form I	Form II	Form I	Form II
1	0–9	3	3	43.96	55.08
2	9–19	33	49	54.46	31.16
3	19–22	53	53	1.58	13.76

Table IV provides a breakdown between the various contributions to the lattice energy highlighting, as expected, the higher lattice energy for the stable form II. Nonetheless, it also reveals that whilst the two forms have similar contribution of vdW interactions to the lattice energy, the % vdW component is much larger (ca. 67%) in form I, whilst form II (ca. 60%) has greater contributions from both H-bond and electrostatic interactions compared to form I. It should be noted that although the form I conformer has a slightly higher molecular polarity, the polar functional groups in the form II crystal structure approach at a much closer distance than in form I in order to maximize hydrogen bonding efficiency. Hence, this is reflected in the larger H-bonding and coulombic components of form II's lattice energy.

**Table IV:** Details of the relative contribution of vdW, coulombic energy and H-bond energy to the total lattice energy of ritonavir forms I and II, highlighting the higher vdW contribution to for form I with respect to form II and vice versa for coulombic energy

Type	Form I	Form II	Percentage contribution to lattice energy %	
			Form I	Form II
vdW (kcal/mol)	−53.77	−55.55	68.6	60.2
Coulombic forces (kcal/mol)	−11.39	−18.54	14.5	20.1
H-bond (kcal/mol)	−13.13	−18.24	16.8	19.8
Lattice Energy (kcal/mol)	−78.29	−92.33	100	100

#### Relative Contributions of the Molecular Fragments to the Lattice Energy

**Fig. 7:**
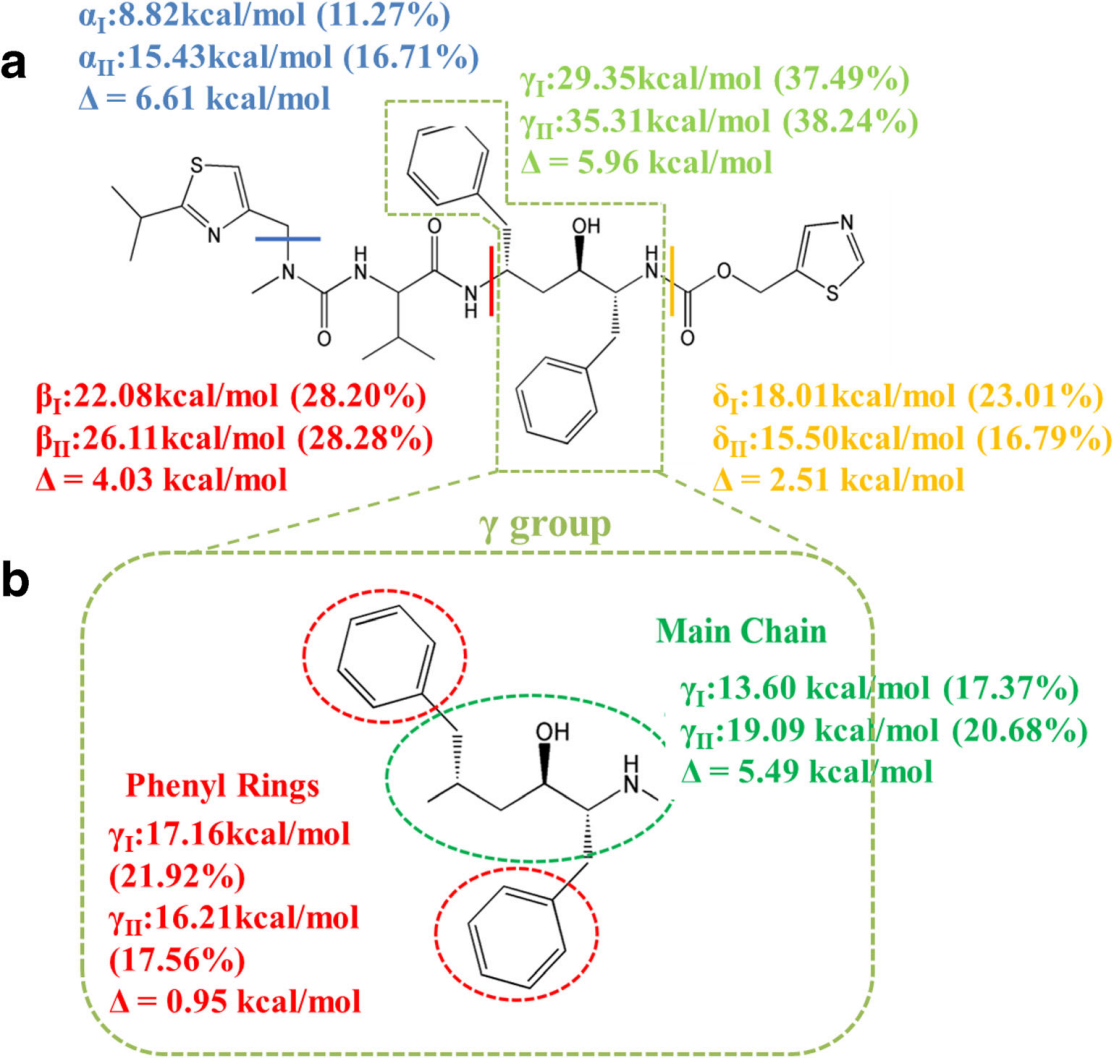
Molecular structure highlighting: (a) the absolute energetic and relative contributions of the four constitutive molecular fragments α, β, γ and δ to the overall lattice energy of ritonavir forms I and II; (b) a more detailed breakdown of the γ fragment highlighting the increased importance of the H-bonding group in form II.

Figure 7 shows the contribution of the different molecular fragments to the lattice energies of forms I and II. The α and δ fragments, containing the thiazole functionalities, make distinctly different contributions in form I (11.27% and 23.01% respectively) and form II (16.71% and 16.79% respectively) reflecting the δ group’s S atom being a hydrogen bond acceptor only in form I. Examination of the β fragment, containing the N-methyl urea functional group, reveals quite similar contribution the lattice energy for both forms (28.2% and 28.28% respectively). Whilst this is apparently the same for the γ group containing the hydroxyl group and the two phenyl rings (37.40% and 38.24%, respectively), closer examination shown in Fig. 7 (b) reveals interesting differences between the functional groups within these molecular fragments. These reflect stronger hydrogen bonding in form II where the cis conformation of the carbamate group rotates the adjacent phenyl group away from the hydroxyl group thus enabling it to act as both a donor and acceptor in contrast to form I where its access is more constrained and hence where it can act as a donor. In contrast, the trans conformation of the carbamate group enables a higher degree of close packing of the phenyl rings in the form I structure but in doing so restricts opportunities for the hydroxyl group to achieve its optimal hydrogen bonding configuration.

#### Intrinsic Synthon Chemistry

Table V (molecular structures in supporting information S5) shows the most important intermolecular interactions for the two forms revealing hydrogen bonding interactions (synthons A_I_ and B_I_ in form I and synthon A_II_ in form II, see further detail in supporting information S5) to have the highest interaction energies, contributing to more than 54% and 62% of the lattice energy respectively.

**Table V:** Details of the five most important synthons in the crystal structures of ritonavir forms I and II as ranked by synthon strength together with their contributions in multiplicity to the dominant crystal habit planes (hkl) important in the crystal morphology

Form I Synthons	Multiplicity	vdW Energy (kcal/mol)	H-bond Energy (kcal/mol)	Coulombic Energy (kcal/mol)	Total Intermolecular Energy (kcal/mol)	Contribution to the Lattice Energy %	Intermolecular Interaction Type	Side faces			Capping face
								{0 0 1}	{1 0 0}	{1 0–1}	{0 1 1}
**A**_**I**_	**2**	**−9.15**	**−4.62**	**−3.4**	**−17.17**	**43.96**	**H-Bond**	**0**	**0**	**0**	**2**
**B**_**I**_	**2**	**−4.72**	**−2.00**	**−0.6**	**−7.32**	**18.70**	**H-Bond**	**0**	**0**	**0**	**1**
C_I_	2	−2.55	–	−0.65	−3.2	8.59	vdW	0	2	2	1
D_I_	2	−2.52	–	−0.58	−3.1	8.32	vdW	0	2	2	0
E_I_	2	−1.62	–	−0.58	−2.2	5.91	vdW	2	2	0	1
Total						83.59					
Form II Synthons	Multiplicity	vdW Energy (kcal/mol)	H-bond Energy (kcal/mol)	Coulombic Energy (kcal/mol)	Total Intermolecular Energy (kcal/mol)	Contribution to the Lattice Energy %	Intermolecular Interaction Type	Side faces		Capping faces	
								{0 1 1}	{0 0 2}	{1 0 1}	{1 1 0}
**A**_**II**_	**2**	**−9.66**	**−9.11**	**−6.40**	**−25.17**	**54.54**	**H-Bond**	**0**	**0**	**2**	**2**
B_II_	2	−4.61	–	−1.42	−6.03	13.06	vdW	2	4	2	2
C_II_	2	−2.38	–	−0.49	−2.87	6.217	vdW	4	0	0	4
D_II_	2	−2.07	–	−0.27	−2.34	5.069	vdW	2	4	2	2
E_II_	2	−1.93	–	−0.05	−1.98	4.29	vdW	2	4	2	2
Total						83.18					

The molecules in synthon A_I_ are related through *b* axis translational symmetry with a repeat of 5.215 Å whereas in synthon A_II_ the molecules are related by screw axis along the *a* axis to give the 9.831 Å short axis repeat. Interestingly, in the form I structure, synthon B_I_ links the molecules through the screw axis and this, in combination with synthon A_I,_ creating two chains of interactions in the same orientation along the *b* axis consistent with the polar nature of this crystal structure. Conversely in form II, the H bond chains are not linked together and hence can run freely in opposite directions along the crystallographic *a* axis, which is more consistent with its higher P2_1_2_1_2_1_ symmetry. Additional detail concerning the hydrogen bonding motifs are provided in the supporting information S6.

The combined interaction energy for the A_I_ and B_I_ H-bonding synthons in form I is significantly less (−22.63 kcal/mol) than that for synthon A_II_ of form II (−25.18 kcal/mol). This reflects the hydrogen bond contribution provided by the hydroxyl group being much stronger in form II compared to form I reflecting that it acts only as a hydrogen bond donor in form I whereas it acts as both a donor and acceptor in form II. The remaining intermolecular interactions are due to weaker and less directed van der Waals interactions and comparatively are very similar for both forms and, hence, the major differences between the synthonic structures of these two polymorphs was found in Synthons A_I_, A_II_ and B.

Table VI provides a more detailed breakdown of the constituent hydrogen bonds associated with synthons A_I_, B_I_ and A_II_ revealing that both polymorphs have 4 different hydrogen bonds that utilize the same hydrogen bonding donors albeit with some differences in terms of which of the 11 acceptor sites are accessed. In this, form I has two synthons:

**Table VI:** Detailed analysis at the atomic level of the constituent H-bonding interactions involved in the three H-bonding synthons identified in Table V highlighting the geometrical details of the contribution donor (D) and acceptor (A) sites together with their respective polarisability. The hydrogen atom in the hydroxyl group of form I is in the same position as used to calculate the lattice energy of the material, for clarity the oxygen atoms are labelled as O = and O- to denote the double or single bonded oxygen to carbon environment respectively

Synthons	H-Bonds Multiplicity	D-H···A	q_D_ /ecu	q_A_ /ecu	q_diff_ /ecu	H···A /Å	D···A /Å	D-H···A /°
A_I_ (Form I)	3	N-H (Amide)···O = (Amide)	0.2243	−0.3915	0.6158	2.149	3.297	153.08
		N-H (N-Methyl Urea)···O = (N-Methyl Urea)	0.2225	−0.4305	0.6530	2.406	3.275	157.5
		N-H (Carbamate)···O = (Carbamate)	0.2236	−0.4216	0.6452	2.199	3.100	154.55
B_I_ (Form I)	1	O-H (Hydroxyl)···N (Thiazole 2)	0.1986	−0.1068	0.3054	2.165	3.131	168.99
A_II_ (Form II)	4	N-H (Amide)···O- (Hydroxyl)	0.2184	−0.3121	0.5305	2.087	3.022	162.52
		N-H (N-Methyl Urea)···O = (Carbamate)	0.2221	−0.4316	0.6537	2.095	3.016	159.39
		N-H (Carbamate)···O = (Amide)	0.2525	−0.3788	0.6313	1.979	2.883	134.0
		O-H (Hydroxyl)···O = (N-Methyl Urea)	0.2001	−0.4379	0.6380	2.033	2.921	152.46

A_I_ comprising 3 hydrogen bonds;

- N-H (Amide)···O = (Amide);
- N-H (N-Methyl Urea)···O = (N-Methyl Urea);
- N-H (Carbamate)···O = (Carbamate)

B_I_ having 1 hydrogen bond;

- O-H (Hydroxyl)···N (Thiazole 2)

Form II has a single synthon:

A_II_ comprising 4 hydrogen bonds;

- O-H (Hydroxyl)···O = (N-Methyl Urea);
- N-H (N-Methyl Urea)···O = (Carbamate);
- N-H (Carbamate)···O = (Amide);
- N-H (Amide)···O- (Hydroxyl)

In comparison, form I has 1 OH-N and 3 different NH-O interactions and whilst form II has one OH-O and 3 different NH-O interactions. Essentially, the hydroxyl interaction in form I changes from OH-N to the much stronger OH-O bond in form II, the latter is, in addition, also stronger due to the cooperative effects of the hydroxyl group acting as both donor and acceptor. Despite the fact that the metastable form I has a more close-packed structure, with a higher density, than the stable form II, its hydrogen bonding is much weaker as evidenced by hydrogen/acceptor distances that are ca. 7% longer than those in form II. The hydrogen bonds in the form I structure were found to be 2.149–2.406 Å, significantly longer than those found in the form II structure which were 1.979–2.095 Å This indicates that although the overall crystallographic packing is less dense, the deformed conformation of the form II molecule in the solid state allows a much closer approach of the optimal hydrogen bond acceptor and donor atoms in the crystal lattice.

### Surface Properties

**Fig. 8:**
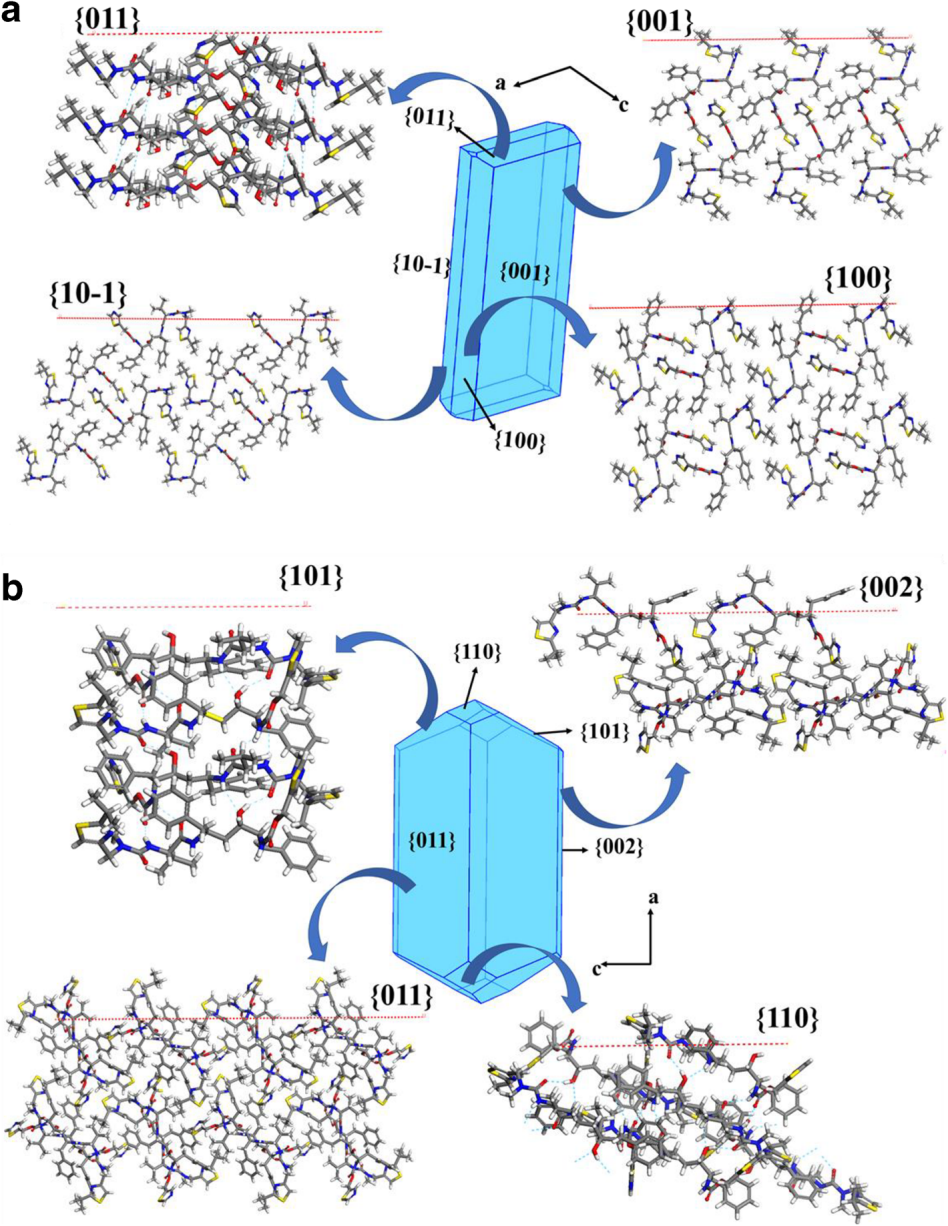
(a) Predicted crystal morphologies for form I and (b) form II (bottom) highlighting the expected surface chemistry of the morphologically important habit faces.

**Fig. 9:**
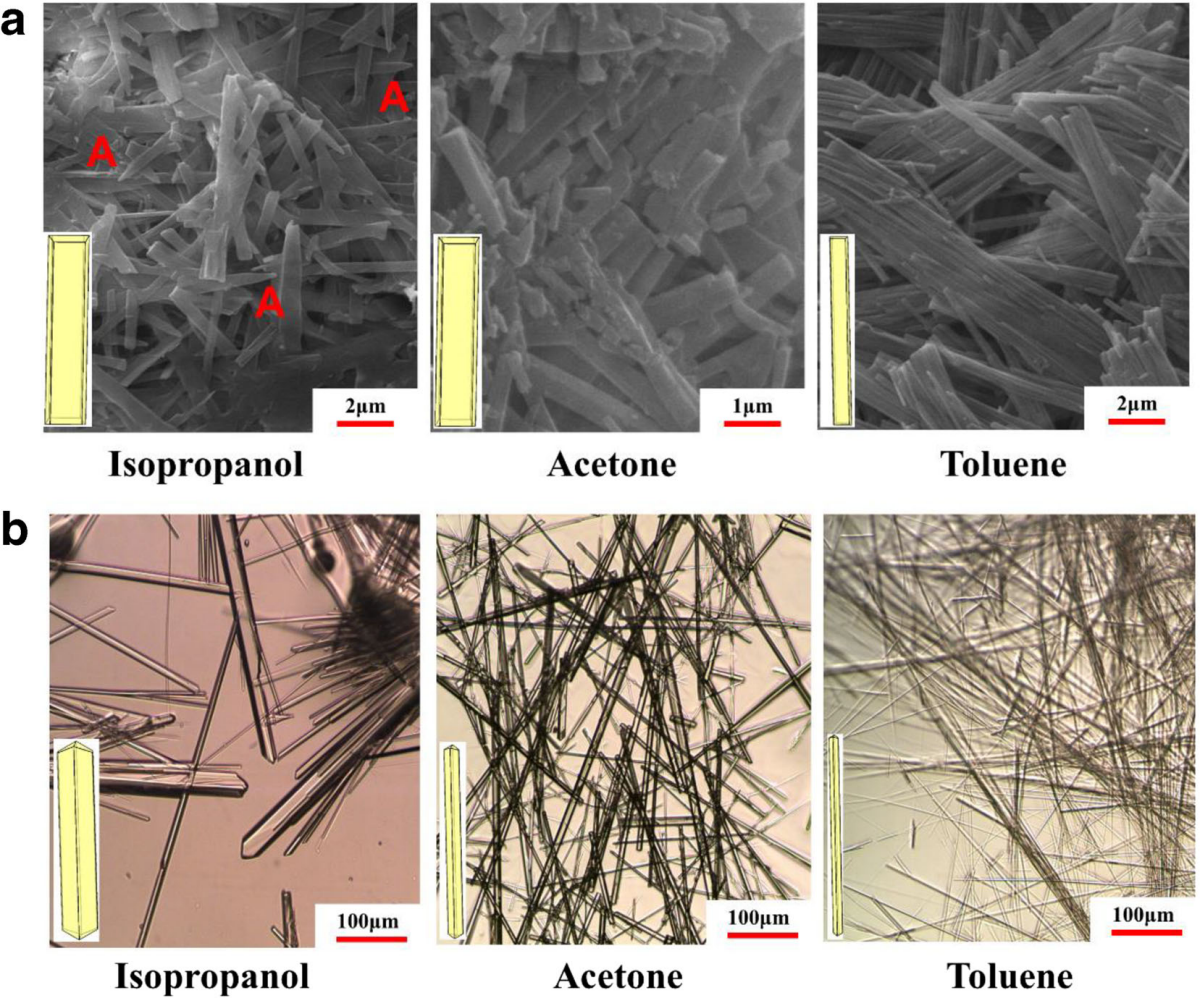
SEM and optical micrographs (respectively) of the observed morphologies as a function of crystallization solvent used together with associated morphological sketches (inset) of (a) form I and (b) form II. It is noteworthy that some crystals prepared from the polar solvents (labelled A) show evidence of some tapering consistent with a polar morphology.

**Fig. 10:**
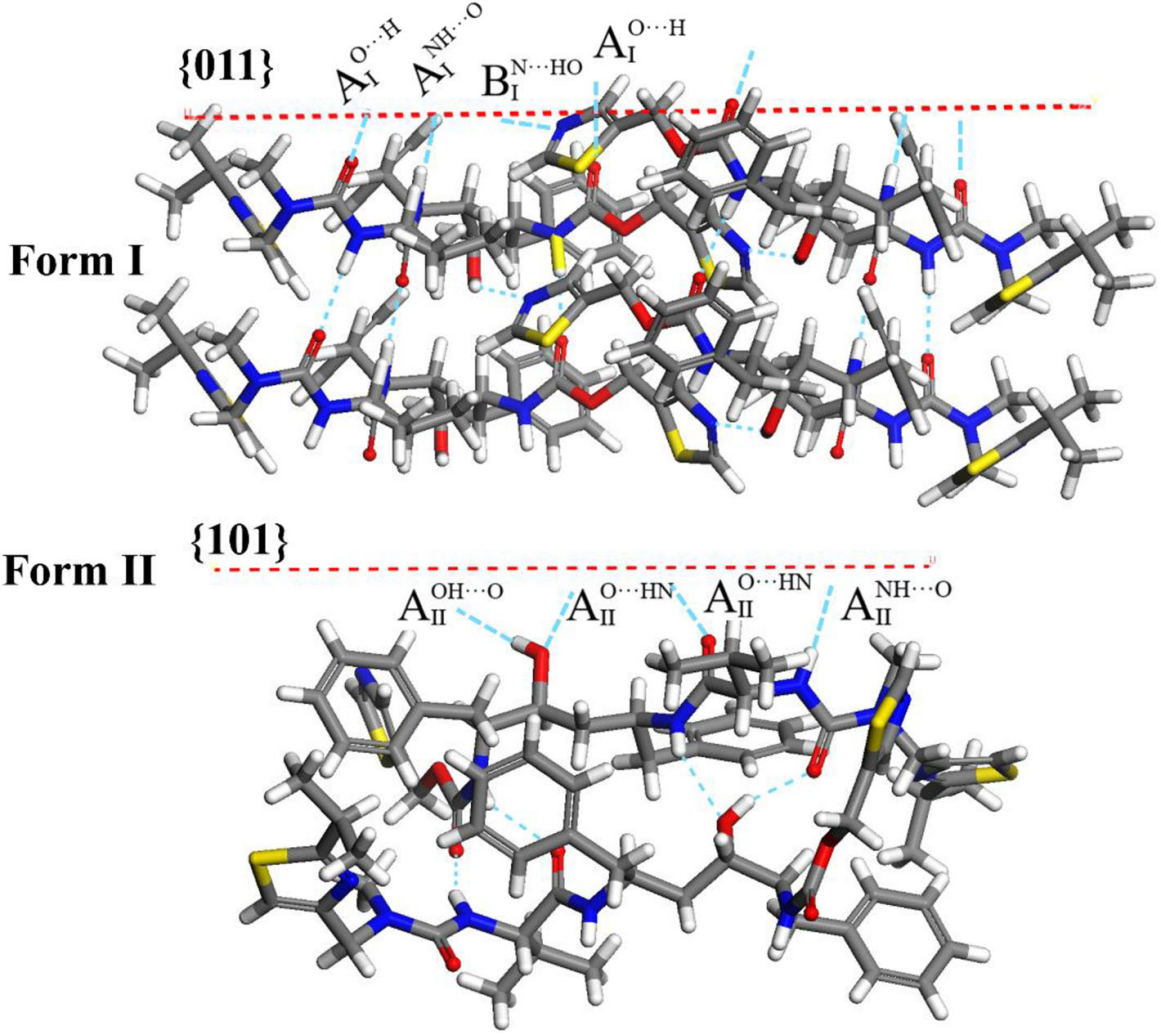
Details of the surface chemistry of the {0 1 1 and {1 0 1} capping face for forms I (a) and II (b) respectively which highlight the inter-atomic hydrogen bonds and their directionality associated with the A_I_, A_II_ and B_I_ synthons. It is noteworthy that B_I_ does not significantly contribute to the growth of the capping faces of form I.

The results of the 3D morphological simulations together with the associated surface chemistry of the dominant crystal habit faces for the two polymorphs is given in Figs. 8 and 10 and Table VII with further information being provided in detailed synthon analysis given in Table V and in the supplementary materials (see sections S5 and S6). Associated experimental data is provided in Fig. 9.

**Table VII:** Calculated attachment energies together with the degree of surface synthon saturation, surface energies, the latter in terms of their breakdown in terms of vdW, hydrogen bonding and electrostatic contributions for the important morphological faces (hkl) for forms I and II

Crystal Surface	d-Spacing / Å	Surface Area / %	Multiplicity	E_att_^hkl^ (kcal/mol)	ξ_hkl_ %	vdW Surface Energy (mJ/m^2^)	H-bond Surface Energy (mJ/m^2^)	Electrostatic Surface Energy (mJ/m^2^)	Total Surface Energy (mJ/m^2^)
**From I**									
{0 0 1}	25.96	58.72	**2**	−9.87	87.39	89.75	–	8.87	98.62
{1 0 0}	12.98	15.08	**2**	−22.30	71.53	93.27	–	18.08	111.35
{1 0–1}	12.87	14.68	**2**	−22.19	73.18	98.21	–	11.69	109.90
{0 1 1}	5.11	11.32	**4**	−57.29	26.82	73.82	21.86	16.91	112.59
Average (whole crystal) surface energy				**−18.90**	**75.88**	**89.54**	**2.47**	**11.57**	**103.58**
**Form II**									
{0 1 1}	13.65	58.48	**4**	−27.01	70.75	116.48	–	22.51	138.99
{0 0 2}	10.13	12.52	**2**	−29.23	68.34	93.69	–	18.12	111.81
{1 0 1}	8.84	25.4	**4**	−42.43	54.04	51.95	58.09	25.24	135.28
{1 1 0}	8.68	3.56	**4**	−50.66	45.13	76.51	60.17	30.62	167.30
Average (whole crystal) surface energy				**−32.04**	**65.26**	**95.77**	**16.90**	**22.93**	**135.60**

#### **Crystal Morphology and Surface Chemistry of Form I**

The predicted morphology, summarized in Fig. 8 (a) and Table VII, reveals an elongated plate-like morphology dominated by {0 0 1}, {1 0 0}, {1 0–1} and {0 1 1} crystal habit surfaces in broad agreement with the experimental morphology in Fig. 9 (a). The form I structure, space group P2_1_, has a polar b-axis which is capped at one end by two {0 1 1} faces and at the other end by two {0–1 1} faces. These crystallographically inequivalent forms are treated as equivalent by the predictive methods used in this study (see further discussion later regarding this).

Examination of the anisotropy factors in Table VII reveals that the top three morphologically important prism surfaces, {0 0 1}, {1 0 0} and {1 0–1}, have a high degree of surface saturation of their intermolecular interactions, 86.7%, 70.1% and 70.2%, respectively. This would suggest that the growth of these surfaces being relatively slow due to the low number of unsaturated interactions available to promote growth and hence a low solute attachment rate. In contrast, the faster growing {0 1 1} capping surfaces have a very much lower surface saturation 26.8% highlighting significant surface bonding opportunities being available at the growth interface to encourage molecular attachment.

The synthon analysis summarized within Table V shows how the five strongest extrinsic inter-molecular synthons contribute to the attachment energies for the {0 0 1}, {1 0 0}, {1 0–1} and {0 1 1} habit surfaces. The data reveals that only the comparatively weak vdW synthon E_I_ contributed to the {0 0 1} surface attachment energy consistent with this surface being slow growing and hence of high morphological importance. Examination of the two sides faces {1 0 0} and {1 0–1} within the b-axial zone reveals only vdW synthons (C_I_, D_I_ and E_I_, and C_I_ and D_I_, respectively) contributed to their surface attachment with albeit with a greater total interaction energy correlating well with these being growing faster faces with a lower morphological importance than {0 0 1}. However, none of attachment processes for these surfaces contained the strongest hydrogen bonding synthons A_I_ and B_I_ seen in form I which were only exposed on the capping {0 1 1} surfaces. In summary, the external morphology of form I crystals can be seen to be characterized by strong growth due to hydrogen bonding along the b-crystal axis complemented by much weaker intermolecular binding through vdW interactions on the plate and side faces. Overall, this is consistent with the formation of elongated plate-like crystals of form I (see further detail in the supporting information, S7).

#### **Crystal Morphology and Surface Chemistry of Form II**

The predicted morphology, summarized in Fig. 8 (b) and Table VII (bottom), reveals an elongated prismatic crystal habit dominated by {0 1 1}, {0 0 2}, {1 0 1} and {1 1 0} crystal habit surfaces, in broad agreement with the observed experimental morphology given in Fig. 9(b). It noteworthy that the predicted morphology encompassing the {h k 0}, {h 0 l} and {0 k l} forms is rather typical of other P2_1_2_1_2_1_ structures where there is a lowering of the morphological importance of the principle axis planes {1 0 0}, {0 1 0} and {0 0 1} surfaces due to the growth slice replication effected by the three 2_1_ screw axes^18^.

Examination of the anisotropy factors in Table VII reveals that the {0 1 1} and {0 0 1} prism faces, the former of which are the morphologically most important surfaces, have a high degree of surface saturation of their intermolecular interactions, 70.8% and 68.3%, respectively. This reflects the low number of unsaturated interactions available to promote growth and hence a low solute attachment rate and hence implies quite a slow growth rate for these surfaces. Nonetheless, these are lower than those for form I. Additionally, the faster growing {1 0 1}, {1 1 0} capping faces have only a slightly lower degree of surface saturation, 54.0% and 45.1%, respectively, than the prism faces, consistent with higher surface bonding opportunities being available at the growth interface to encourage molecular attachment and growth. Overall, these data suggests a higher growth rate for form II when compared to form I.

Examination of the top five synthons for form II with respect to the predicted attachment energies in Table VII, reveal the capping {1 1 0} and {1 0 1} surfaces to be only habit planes whose surface binding involved the strongest and hydrogen-bonding extrinsic synthon A_II_. In contrast, the larger {0 1 1} and {0 0 2} prism faces do not involve any contribution from synthon A_II_ with their growth being promoted by the weaker and less directional vdWs interactions (synthons B_II_, C_II_, D_II_ and E_II_ and synthons B_II_, D_II_ and E_II,_ respectively) consistent with a much slower molecular attachment process at the growth interface with concomitantly higher surface areas for the prism habit forms. Visualization of the surface chemistry for the observed habit surfaces, shown in Fig. 8(b), reveals the contrasting surface chemistry of hydrophobic {0 1 1} and {0 0 2} prism faces and the {1 0 1} and {1 1 0} hydrophilic capping faces. The latter is shown in closer detail in Fig. 10(a) and (b), respectively, highlighting their closed-packed in-plane structure characterized by the exposed hydrogen bond donor and acceptor sites which facilitate formation of the energetically favorable strong intermolecular synthon A_II_ (see further details in section S8 of the supporting information).

#### Comparing the Crystallization Behavior of Forms I and II

The experimentally observed crystal morphologies of forms I and II, following re-crystallization in isopropanol (protic), acetone (aprotic), and toluene (apolar) solvents, are shown in Fig. 9(a) and (b) respectively. The observed morphologies of both forms were found to be more needle-like when compared to predicted morphologies. This effect, which was found to be noticeably stronger for the form II crystals, reflects the higher growth-promoting ‘reactivity’ of the hydrophilic capping faces as evidenced by their strong H-bonding propensity and much lower degree of surface saturation which, under kinetic growth conditions, would hence be expected to result in a faster growth rate.

The form I crystals grown from the polar solvents isopropanol and acetone showed no noticeable differences between their aspect ratios, probably due to the hydrophilic capping surfaces exposing H-bond binding sites e.g. on the {0 1 1} capping surface (see Fig. 8) hence hindering its growth through the selective binding of these two solvents. In contrast, crystals grown from the apolar toluene solvent had significantly higher aspect ratios than those from the more polar solvents consistent with the formation of strong π-π solvation interactions between the phenyl ring in toluene and the aromatic rings exposed on the hydrophobic {1 0–1} and {1 1 0} side crystal surfaces (see Fig. 8). Such solvent binding would be expected to disrupt the growth of these surfaces but would have very little influence on the growth of the more polar capping faces with the overall effect of making these crystals more needle-like. Interestingly, close examination of the morphological data reveals some tapering along the b-axis (labelled A) providing evidence for the development of a polar morphology from the polar solvents.

Examination of the form II crystals broadly mirrored that for the form I crystals albeit the solvent dependence of these morphologies was much stronger with the aspect ratio increasing in the order of isopropanol, acetone and toluene respectively consistent with their respective abilities to bind to the H-bond donor and acceptor groups exposed on e.g. the {1 0 1} capping surface (see Fig. 8). Isopropanol, as a protic solvent with both H-bond donor and acceptor sites within its hydroxyl group, can be expected to have the strongest binding to these hydrophilic surfaces hence competing with the formation of extrinsic synthon A_II_ and effectively acting as a retarder to the growth of these faces. This growth-inhibiting effect would be expected to be much weaker for the aprotic acetone which only has H-bond acceptor capabilities whilst the apolar toluene molecules would be more likely to preferentially bind to the hydrophobic {0 1 1} and {0 0 2} prism faces which would be consistent with it having a much higher aspect ratio.

A more detailed quantitative analysis seeking to compare the crystallization kinetic behavior of the two forms is quite challenging without having more crystallization data, such as quantitative face-specific growth rate data as a function of supersaturation. Nonetheless, a qualitative comparison reveals a much smaller crystal size distribution for form I than form II which would be consistent with a much higher nucleation rate and greater ease of crystallization for the former. A helpful comparison can be made between crystals grown in isopropanol shown in Fig. 9(a, left) and (b, left) where the crystallization supersaturations were reasonably close (4.1 and 3.0, respectively). The form I crystals were found to be very small in size (ca. 10 μm × 0.5 μm^2^) whilst the form II crystals were much larger in size and to be more elongated (ca. 600 μm × 15 μm^2^). This is consistent with a much higher growth rate for the form II crystals as well as with the predicted attachment energies for the two forms. Overall, this suggests that the rate limiting process for the formation of form II lies in its nucleation rather than growth processes consistent with previous slurry conversion tests (see Fig. 3 Bauer et.al. (1)) i.e. without the presence of seed crystals of form II the primary nucleation is very slow but conversely crystal growth is quite fast with seeding.

#### Whole Crystal Surface Area Weighted Surface Energies

The surface energies for the crystal habit surfaces are given in Table VII; columns 7–10. The whole crystal, surface area weighted, surface energy of form II was found to be larger than that of form I, consistent with this form’s larger lattice energy. However, this difference was found to mostly lie in the polar component as the dispersive surface energy was found to be much the same for both polymorphs. The surface-area weighted crystal surface energies for the predicted crystal habits (Fig. 8) for the two polymorphs following crystallization from the different solvents can also be compared. These showed that crystals formed from polar solvents (e.g. isopropanol, acetone) had a higher surface energy, ca. 105 mJ/m2 for form I and ca. 134 mJ/m2 for form II, when compared to those crystallized from non-polar solvents (e.g. toluene), ca. 104 mJ/m2 for form I and ca. 130 mJ/m2 for form II. Such predictions would be consistent with the expectations i.e. that a decrease in solvent polarity should reduce the surface coverage fraction of the higher energy fast growing reactive faces and hence, resulting in a lowering of the crystal surface energy.^16^

## Conclusions

This work presents a detailed and integrated (mostly computational modelling) analysis, supported by experimental process data, of the molecular and synthonic (inter-molecular) structures that underpin the conformational polymorphism behavior in ritonanvir. This compound is not only an iconic and representative pharmaceutical API but also one which is a well-known example of the potential commercial impact when there is a change in the solid-form of an API within a currently marketed drug product dosage form.

Analysis of the molecular conformation of its two well-known polymorphs, forms I and II, reveal the former has a more stable conformation than form II and that the conformation in the solid-state is only slightly distorted with respect to the minimized energy. Conversely, the conformation in form II was found to be much more distorted with respect to its minimized structure and less stable with a deformation energy of 8.09 kcal/mol compared to that of form I.

Steric hindrance between the conformational states in the two forms would appear to preclude transformation in the solid-state. In its solid-state ritonavir is more efficiently packed with a higher density in form I when compared to form II but in doing so full exploitation of the available high energy hydrogen bonding was not found to be feasible. Notably the central hydroxyl group is shielded by one of the phenyl groups. The enhanced hydrogen bonding afforded by the form II structure yields a significant increase in lattice energy over form I, due to much shorter hydrogen bonding distances, with the former reflecting larger contributions from enhanced polar interactions to its lattice energy. The value of examining and inter-relating the molecular conformation and solid-state intermolecular synthons in the same workflow is realized through the discovery that not only is the form I conformation more stable, consistent with previous studies, but that the form II less stable conformation appears to be necessary to expose a central OH group to form the synergistic hydrogen bonds in form II. Such analysis reveals the delicate balance between conformation and packing in a crystal structure, highlighting the need for further understanding of cases where one is likely to dominate over the other, with respect to predicting the crystallisability of API polymorphs.

Interestingly the form I structure converges its lattice energy at a smaller cluster size, consistent with this form being more likely to form at higher crystallization supersaturations. Fragmentation of the molecular structure regarding their respective contribution to the lattice energy of the two polymorphs, reveals significant differences between the respective contributions from both the terminal thiazole groups and the central aromatic OH hydrogen bonding groups.

Detailed characterization of the intermolecular (synthons) interactions revealed an overarching structure of mostly 1-D anisotropic hydrogen bonding coupled with strong van der Waals interactions. The strongest hydrogen bonding differences between the form I and II structures were found to be quite subtle with four NH-O interactions within two synthons (A_I_ as B_I_) in form I against an OH-O and three NH-O interactions in a single synthon (A_II_) in form II.

The structural anisotropy was found to impact strongly on the crystal morphology of the two forms with the hydrogen bonding mostly propagating along a single axis (*b* in form I and *a* in form II), resulting in elongated crystal habits, tabular in form I and prismatic in form II. In both forms the surface synthon saturation is weak on the capping faces and this was found to lead to further elongation in crystals when grown from supersaturated solutions. Whilst this effect was found to be less pronounced in protic solvents when solvent binding to the hydrogen bonded surfaces was found to reduce the crystal growth, in apolar solvents very large (>100) aspect ratio crystals were observed. Comparison of the overall crystal properties revealed the surface area weighted crystal surface energy to be, as expected, higher for form II albeit with similar dispersive but much larger polar surface energy components.

Overall, the paper highlights the value of a rigorous and comprehensive crystallographically-based workflow and analysis of a challenging and highly representative pharmaceutical material which displays known conformational polymorphic behavior. In this, the work provides an assessment of physical-chemical properties of the polymorphic forms, highlighting the interrelationship between molecular, solid-state and surface structures.

### Acknowledgments and Disclosures

One of us (CW) acknowledges funding support from the China Scholarship Council for a visiting scholarship at Leeds. This work was also supported by the ‘Advanced Digital Design of Pharmaceutical Therapeutics’ (ADDoPT) project (AMSCI Grant No. 14060) and builds upon previous work on morphological modelling (EPSRC grant EP/I028293/1 and crystallization (EPSRC grant EP/IO14446/1 and EP/IO13563/1) research at Leeds, the latter in collaboration with the University of Manchester. AbbVie provided the crystalline source material; contributed to the design of the study and workflow; were involved in the interpretation of data; assisted draft paper reviewing; and in the approval of the final submitted publication. One of us (AS) is an employee of AbbVie and he may own AbbVie stock.

## Supplementary Information

ESM 1 (DOCX 3643 kb)

## List of Symbols

∆*H*_*f*_: Enthalpy of fusion
*E*_*cr*_: Lattice energy
${E}_{sl}^{hkl}$: Slice energy per surface hkl
${E}_{att}^{hkl}$: Attachment energy per surface hkl
ξ_hkl_: Surface anisotropy factor
γ_particle_: Particle surface energy
*γ*_*(hkl)*_: Surface energy of a given crystallographic face
*M*_*(hkl)*_: Multiplicity of the habit face
*V*_*cell*_: Volume of the crystallographic unit cel
*Z*: Number of molecules in the unit cell
*d*_*hkl*_: Inter-planar d-spacing
*N*_*A*_: Avogadro’s number
*SA*_*(hkl)*_: Fractional surface area of the habit face (hkl)
Å: Angstroms
*τ*: Interatomic torsion angle of molecular fragmen
RMSD: Root-mean-square deviation
