# Supplementary material for: Molecular, Solid-State and Surface Structures of the Conformational Polymorphic Forms of Ritonavir in Relation to their Physicochemical Properties
Source: Pharm Res. 2021 May 19;38(6):971–90. doi: 10.1007/s11095-021-03048-2 (PMC8217055; doi:10.1007/s11095-021-03048-2)
Supplement: Supplementary file 1 — (DOCX 3643 kb) [file 11095_2021_3048_MOESM1_ESM.docx]

**Molecular, Solid-State and Surface Structures of the Conformational Polymorphic Forms of Ritonavir in Relation to their Physicochemical Properties**

*Chang Wang^1,2^, Ian Rosbottom^2^, Thomas D. Turner^2^, Sydney Laing^2^, Andrew G. P. Maloney^3^; Ahmad Y. Sheikh^4^, Robert Docherty^†2^, Qiuxiang. Yin^1*^ and Kevin J. Roberts^2*^*

*^1^ School of Chemical Engineering and Technology, State Key Laboratory of Chemical Engineering, Tianjin University, China.*

*^2^ Centre for the Digital Design of Drug Products, School of Chemical and Process Engineering, University of Leeds, Woodhouse Lane, Leeds, LS2 9JT, UK.*

*^†^ Visiting Professor of Pharmaceutical Science and Engineering, previously at Pfizer, Sandwich, UK*

*^3^ The Cambridge Crystallographic Data Centre, 12 Union Road, Cambridge, CB2 1EZ, UK.*

*^4^ Solid State Chemistry, Process R&D, AbbVie Inc., North Chicago, IL, 600645, USA.*

* Corresponding Author

**S1. Details of Habit98 Lattice Energy Calculations**

**Form 1.**

PROGRAM OPERATING IN LATTICE ENERGY MODE

UNIT CELL PARAMETERS: A = 13.34000 ANGSTROMS

B = 5.21000 ANGSTROMS

C = 26.69000 ANGSTROMS

ALPHA = 90.00000 DEGREES

BETA = 103.46000 DEGREES

GAMMA = 90.00000 DEGREES

THE BONDING ANALYSIS MODE (DM) IS 40

CSSR FILE OF CHOSEN BOND FOR ALL ALLOWED FACES/RADII/SHIFTS/ORIGINS

------------ Inputted Structural Data ------------

NUMBER OF ATOMS IN THE ASYMMETRIC UNIT IS 98

NUMBER OF ASYMMETRIC UNITS IN THE UNIT CELL IS 2

NUMBER OF MOLECULES IN THE ASYMMETRIC UNIT IS 1

--------- Potential Function Information ---------

Potential set chosen is Dreiding II, combining a

6-12 potential with a 10-12 H-bond potential, and

with parameters from S.L. Mayo, B.D. Olafson and

W.A. Goddard III, J. Phys. Chem. 1990 (94) 8897.

Also includes the 6-exp potential with parameters

from D.E. Williams, J. Chem. Phys. 45 (1966) 3370,

and the 6-exp potential with parameters from A.

Rohl et al. (1993).

------------- Lattice Energy Results -------------

LIMITING RADIUS = 30.00 ANGSTROMS

LATTICE ENERGY = -78.07 KCAL/MOL

ELECTROSTATIC INTERACTION = -11.44 KCAL/MOL

--------------------------------------------------

**Form 2.**

PROGRAM OPERATING IN LATTICE ENERGY MODE

UNIT CELL PARAMETERS: A = 9.83000 ANGSTROMS

B = 18.48000 ANGSTROMS

C = 20.26000 ANGSTROMS

ALPHA = 90.00000 DEGREES

BETA = 90.00000 DEGREES

GAMMA = 90.00000 DEGREES

THE BONDING ANALYSIS MODE (DM) IS 60

CSSR FILE OF CHOSEN BOND FOR ALL ALLOWED FACES/RADII/SHIFTS/ORIGINS

------------ Inputted Structural Data ------------

NUMBER OF ATOMS IN THE ASYMMETRIC UNIT IS 98

NUMBER OF ASYMMETRIC UNITS IN THE UNIT CELL IS 4

NUMBER OF MOLECULES IN THE ASYMMETRIC UNIT IS 1

--------- Potential Function Information ---------

Potential set chosen is Dreiding II, combining a

6-12 potential with a 10-12 H-bond potential, and

with parameters from S.L. Mayo, B.D. Olafson and

W.A. Goddard III, J. Phys. Chem. 1990 (94) 8897.

Also includes the 6-exp potential with parameters

from D.E. Williams, J. Chem. Phys. 45 (1966) 3370,

and the 6-exp potential with parameters from A.

Rohl et al. (1993).

------------- Lattice Energy Results -------------

LIMITING RADIUS = 30.00 ANGSTROMS

LATTICE ENERGY = -92.29 KCAL/MOL

ELECTROSTATIC INTERACTION = -18.57 KCAL/MOL

--------------------------------------------------

**S2. Lipinskis Rule for Ritonavir**

Lipinski's rule states that, in general, an orally active drug has no more than one violation of the following criteria:

- No more than 5 hydrogen bond donors (the total number of nitrogen–hydrogen and oxygen–hydrogen bonds)
- No more than 10 hydrogen bond acceptors (all nitrogen or oxygen atoms)
- A molecular mass less than 500 daltons
- An octanol-water partition coefficient (log P) that does not exceed 5

Note that all numbers are multiples of five, which is the origin of the rule's name.

| **Lipinski’s Rule Criteria** | **Correlation to Ritonavir Structure** | **Lipinski Criteria Met?** |
| --- | --- | --- |
| No more than 5 hydrogen bond donors | 4 | Yes |
| No more than 10 hydrogen bond acceptors | 11 | No |
| Molecular less than 500 daltons | 720.9 | No |
| log P that does not exceed 5 | 5.98 | No |

This lack of any clear alignment with the Lipinski criteria would be consistent with ritonavir’s BMS classification as a class IV drug compound [2], i.e. displaying both low solubility and low permeability.

[1] Lipinski, CA; Lombardo, F; Dominy, BW; Feeney, PJ (March 2001). "Experimental and computational approaches to estimate solubility and permeability in drug discovery and development settings". Advanced Drug Delivery Reviews. 46 (1–3): 3–26.

[2] <https://www.accessdata.fda.gov/drugsatfda_docs/nda/2010/022417s000_ChemR.pdf>

**S3. The Molecular Charge Distribution for the Molecular Conformers of Ritonavir**


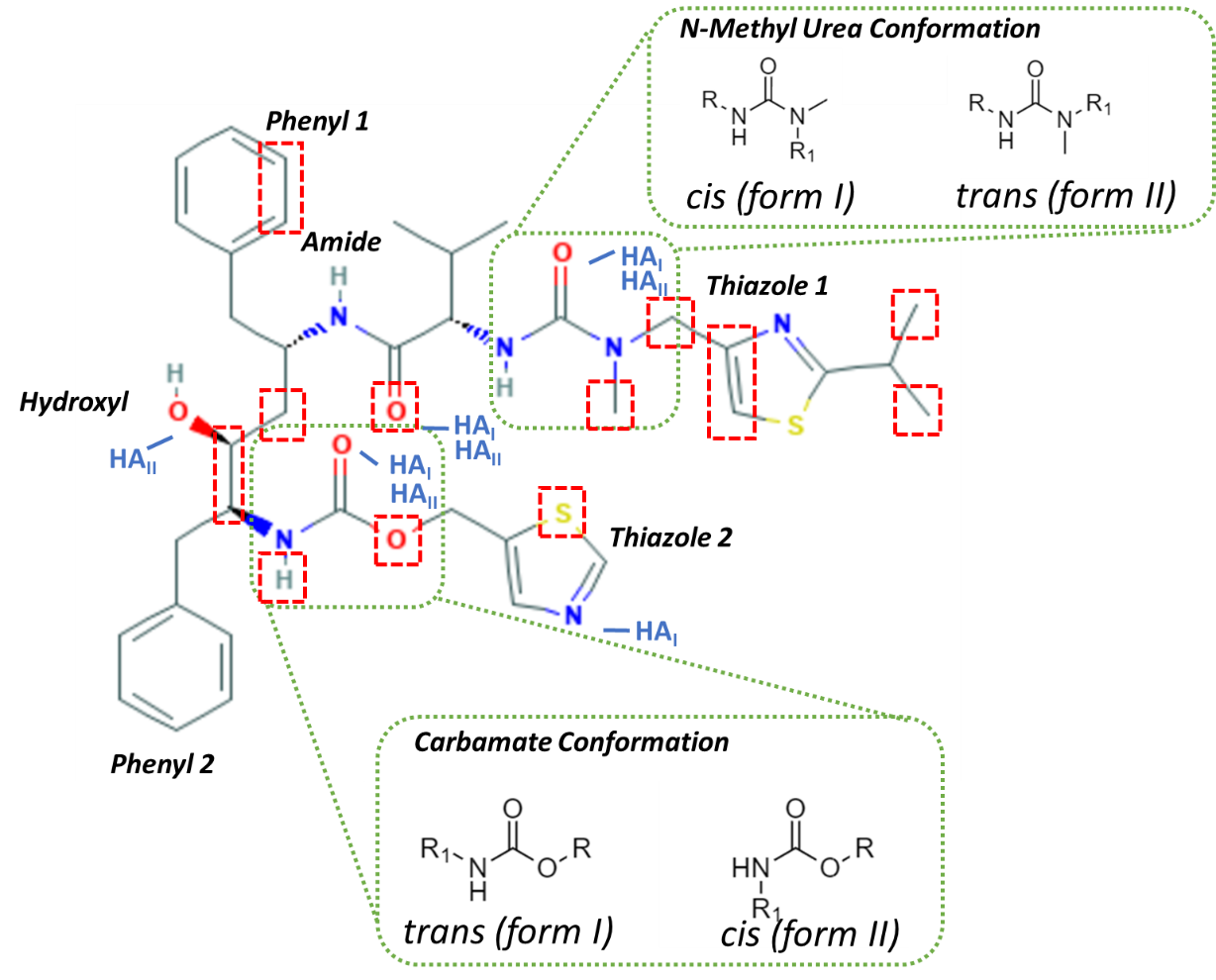


Figure S3.1 Molecular diagram of ritonavir, highlighting the important functionalities and Hydrogen bond acceptors in the molecule together with atoms and groups which show differences in calculated charges between forms I and II are highlighted in red


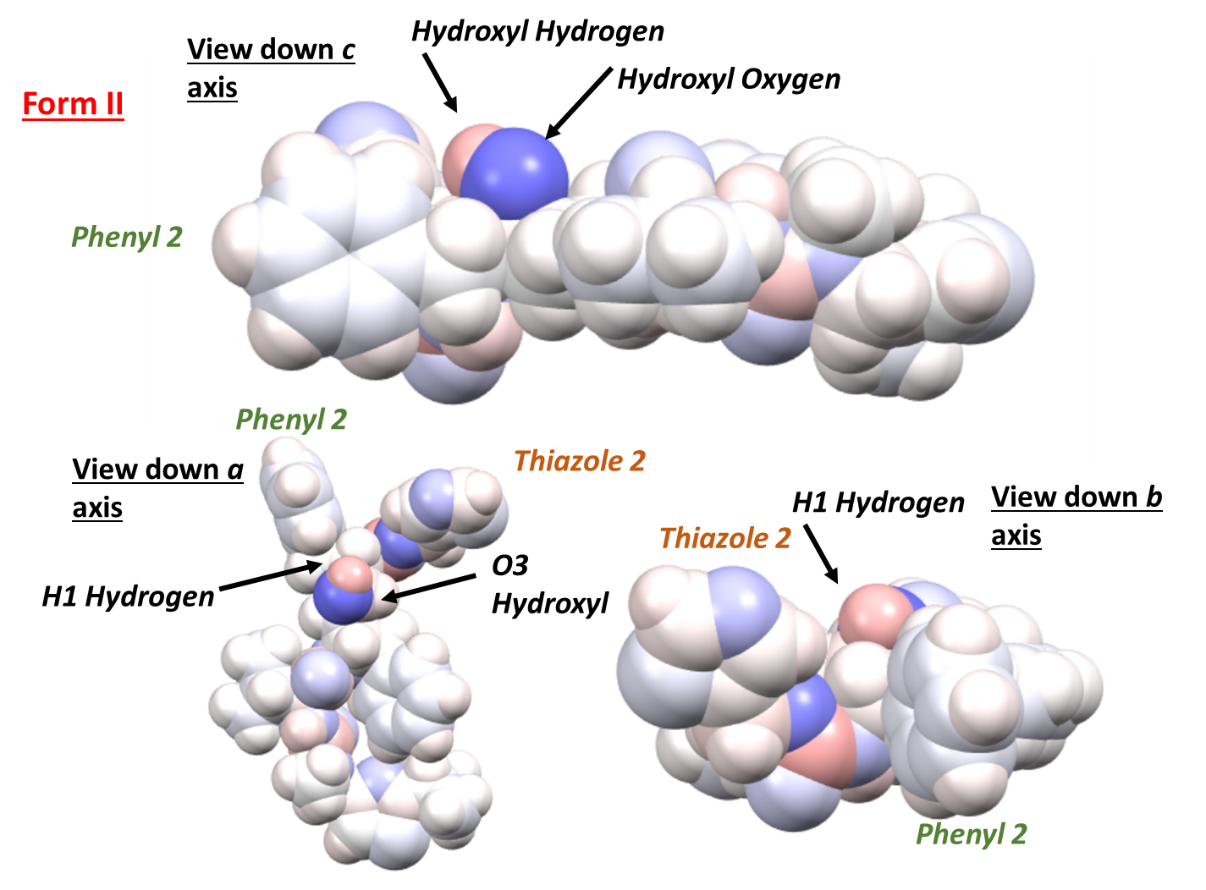


Figure S3.2 Space filling molecular diagram of the ritonavir form II conformer with atoms coloured by Gasteiger atom charge where blue indicates negative polarity and red highlights positive polarity. The important oxygen and hydrogen of the hydroxyl group, O3 and H1 are labelled together with the second phenyl and second thiazole ring as shown previously in Figure 1 and viewed down various crystallographic axes for clarity


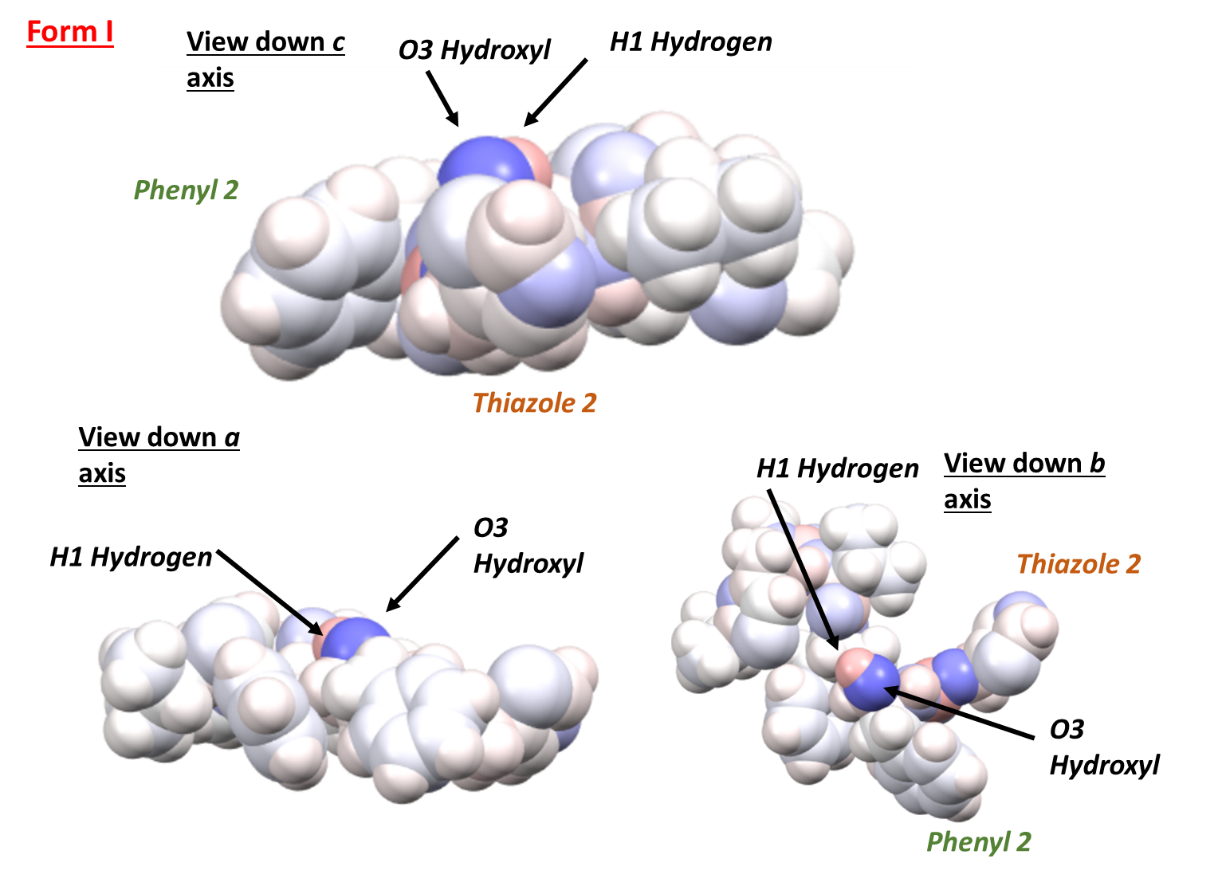


Figure S3.3 Space filling molecular diagram of the ritonavir form I conformer with atoms coloured by Gasteiger atom charge where blue indicates negative polarity and red highlights positive polarity. The important oxygen and hydrogen of the hydroxyl group, O3 and H1 are labelled together with the second phenyl and second thiazole ring as shown previously in Figure 1 and viewed down various crystallographic axes for clarity

The atomic charges calculated for the two ritonavir conformers, form I and II, are provided in Table S2.1 and highlight the differences in calculated atomic charges through Mopac, associated with the conformational change between the two forms. It should be noted that the atom labels and the atom order for the form I molecule were reordered so the atom order matches that in form II for ease of comparison in the table and this discussion, the atom numbers are also provided on the molecular sketch in Figure S2.4. The table also highlights the charge differences between the two forms where these differences are coloured red to blue to highlight positive and negative changes in charge respectively, where the depth of colour represents the magnitude of change, deep colour representing large changes and light colour representing small changes. Significant atomic charge differences are also highlighted in the molecular diagram in Figure S2.1 where atoms with large differences in charge are boxed in red. Analysis of the charge distributions of the ritonavir molecule in both forms reveals that of the 17 polar atoms in the structure (considering here the oxygen, nitrogen, sulphur and their respective hydrogen atoms) 10 of these atoms are more polar in the form I conformation, however only 2 of these ten atoms vary by a significant amount (>0.01 ecu) which include the amide carbonyl oxygen and the carbamate ester oxygen. The analysis also found that 2 of the 4 donor hydrogens in the structure were more polar in form I, these were the n-methyl urea hydrogen and the amide hydrogen. Of the 13 acceptor atoms (including the sulphur atoms for completeness) 8 of these were found to be more polar in the form I conformation. These included 3 of the 5 oxygen atoms namely the amide carbonyl oxygen, the hydroxyl oxygen and the carbamate ester oxygen together with 5 of the 6 nitrogen atoms where only the nitrogen atom of thiazole 1 was found to be less polar in form I. Overall this analysis highlights that the form I conformer is, in general, more polar with respect to the hydrogen bonding atoms within the molecule, albeit with relatively small differences calculated between the two conformers. This difference in polarizability may contribute somewhat to the experimentally determined differences in solubility between the two forms, particularly where form I was found to be much more soluble in polar protic solvent such as alcohols.

The reason for this polarizability difference can be seen in more detail through examination of the molecular conformations for form I and II and through highlighting of the electron density distributions to visually examine the impact of conformational change on these functional groups of interest. The molecular structure of ritonavir is provided in Figure 1 and highlights the important functional groups which surround the hydroxyl group in 3-dimensional space for the two conformers of ritonavir, namely the second thiazole and second phenyl group. Figures S2.2 and S2.3 provide molecular space filling models of the two conformers where the atoms have been coloured in relation to their calculated Gasteiger charge and the functional groups of interest have been highlighted. Figure S2.2 shows that in form II the second thiazole ring and the second phenyl ring are torsioned towards the proton on the hydroxyl group providing shielding of this functionality.

Conversely Figure S2.3 shows the space filling model of the form I conformer where the same thiazole and phenyl functionalities are highlighted to show their position with respect to the hydroxyl group. The two rings in form I are now torsioned away from the hydroxyl group and are positioned away towards the lower part of the molecule with respect to the hydroxyl group. This likely reduces the electronic shielding effect, and this can be seen in the Mopac charge calculations as the hydroxyl was found to be more slightly more polarised in form I. This would mean the proton is more ‘available’ for interaction to, for example, protic solvents for hydrogen bonding from an electronic point of view as the group is more polar.

Overall this analysis highlights that certain polar functionalities show charge density variations between the two conformations of ritonavir, where, in general, form I exhibits a more polarised hydroxyl group. The cause of this is likely to be the torsioning of the second thiazole and second phenyl ring in the structure, away from the hydroxyl group. This has two effects; the first is a decreased shielding effect of the hydroxyl group which is likely the reason for its increased polarity in form I, and secondly the hydroxyl proton is less sterically hindered. These two combined effects may explain in some part the orders of magnitude solubility increase in protic solvents of form I over form II.


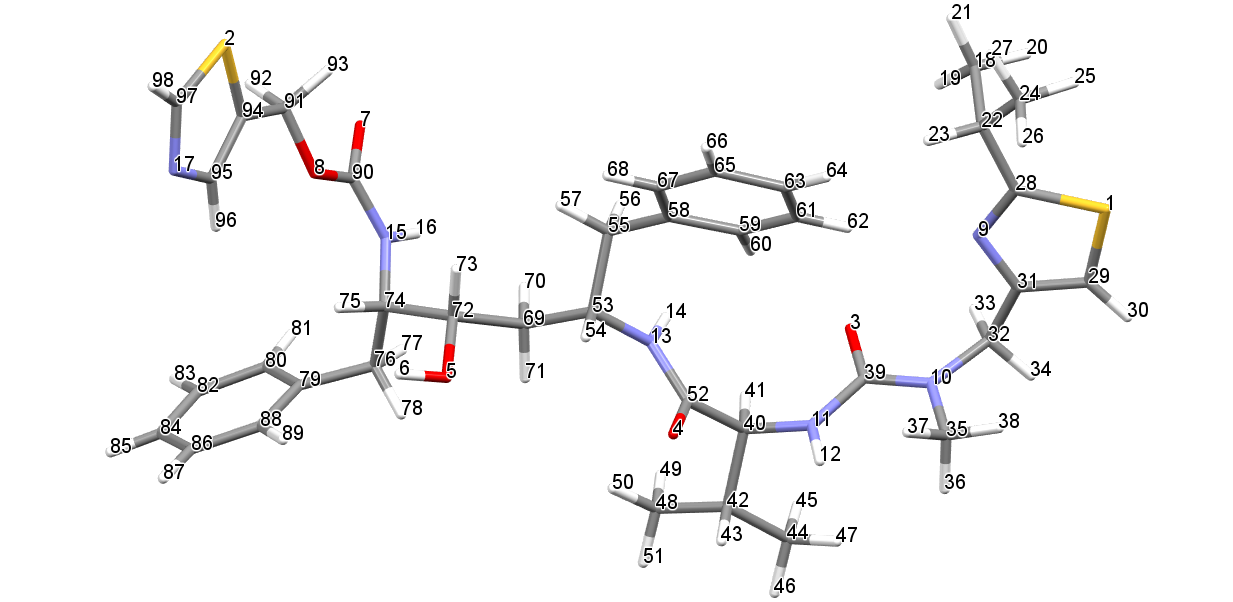


Figure S3.4 Molecular structure of ritonavir to highlight the atom numbering for the purposes of atomic charge comparison

Table S3.1 Atom charges calculated for the molecular conformers of ritonavir forms I and II using MOPAC, the atom-atom charge differences between Form I and II, highlighting the important polar functional groups and their respective functional groups, the polar hydrogens involved in h-bonding are highlighted in green. The first 17 atoms are the polar atoms in the structure.

| **Atom Number** | **Atom Label** | **Form I Mopac Charge (ecu)** | **Form II Mopac Charge (ecu)** | **Charge Difference (ecu)** | | **Functional Group** |  |
| --- | --- | --- | --- | --- | --- | --- | --- |
| **1** | S1 | 0.4593 | 0.4552 | 0.0041 | Thiazole 1 | | |
| **2** | S2 | 0.4839 | 0.4685 | 0.0154 | Thiazole 2 | | |
| **3** | O1 | -0.4305 | -0.4379 | 0.0074 | N-methyl Urea | | |
| **4** | O2 | -0.3915 | -0.3788 | -0.0127 | Amide | | |
| **5** | O3 | -0.3171 | -0.3121 | -0.005 | Hydroxyl | | |
| **6** | H1 | 0.1986 | 0.2001 | -0.0015 | Hydroxyl | | |
| **7** | O4 | -0.4216 | -0.4316 | 0.01 | Carbamate, C=O | | |
| **8** | O5 | -0.3111 | -0.2852 | -0.0259 | Carbamate, C-O | | |
| **9** | N1 | -0.1106 | -0.1147 | 0.0041 | Thiazole 1 | | |
| **10** | N2 | -0.3363 | -0.3322 | -0.0041 | N-methyl Urea, N-methyl nitogen | | |
| **11** | N3 | -0.3743 | -0.3673 | -0.007 | N-methyl Urea, urea nitrogen | | |
| **12** | H2 | 0.2225 | 0.2221 | 0.0004 | N-methyl urea | | |
| **13** | N4 | -0.3598 | -0.3579 | -0.0019 | Amide | | |
| **14** | H3 | 0.2243 | 0.2184 | 0.0059 | Amide | | |
| **15** | N5 | -0.3515 | -0.3435 | -0.008 | Carbamate | | |
| **16** | H4 | 0.2236 | 0.2525 | -0.0289 | Carbamate | | |
| **17** | N6 | -0.1068 | -0.1061 | -0.0007 | Thiazole 2 | | |
| **18** | C1 | -0.2042 | -0.1997 | -0.0045 | Isopropyl 1 | | |
| **19** | H5 | 0.081 | 0.0938 | -0.0128 | Isopropyl 1 | | |
| **20** | H6 | 0.0842 | 0.0718 | 0.0124 | Isopropyl 1 | | |
| **21** | H7 | 0.0717 | 0.0781 | -0.0064 | Isopropyl 1 | | |
| **22** | C2 | -0.0485 | -0.0535 | 0.005 | Isopropyl 1 | | |
| **23** | H8 | 0.0954 | 0.1165 | -0.0211 | Isopropyl 1 | | |
| **24** | C3 | -0.1906 | -0.2094 | 0.0188 | Isopropyl 1 | | |
| **25** | H9 | 0.0878 | 0.0733 | 0.0145 | Isopropyl 1 | | |
| **26** | H10 | 0.0696 | 0.0799 | -0.0103 | Isopropyl 1 | | |
| **27** | H11 | 0.0854 | 0.0855 | -0.0001 | Isopropyl 1 | | |
| **28** | C4 | -0.2541 | -0.2424 | -0.0117 | Thiazole 1 | | |
| **29** | C5 | -0.4121 | -0.4458 | 0.0337 | Thiazole 1 | | |
| **30** | H12 | 0.1706 | 0.1599 | 0.0107 | Thiazole 1 | | |
| **31** | C6 | -0.0947 | -0.0675 | -0.0272 | Thiazole 1 | | |
| **32** | C7 | 0.0615 | 0.0781 | -0.0166 | Backbone | | |
| **33** | H13 | 0.0767 | 0.1381 | -0.0614 | Backbone | | |
| **34** | H14 | 0.1177 | 0.0816 | 0.0361 | Backbone | | |
| **35** | C8 | -0.0426 | -0.0695 | 0.0269 | N-Methyl Urea | | |
| **36** | H15 | 0.0617 | 0.0607 | 0.001 | N-Methyl Urea | | |
| **37** | H16 | 0.078 | 0.0825 | -0.0045 | N-Methyl Urea | | |
| **38** | H17 | 0.109 | 0.0939 | 0.0151 | N-Methyl Urea | | |
| **39** | C9 | 0.4131 | 0.4127 | 0.0004 | N-Methyl Urea | | |
| **40** | C10 | 0.0469 | 0.0488 | -0.0019 | Backbone | | |
| **41** | H18 | 0.1279 | 0.1214 | 0.0065 | Backbone | | |
| **42** | C11 | -0.112 | -0.1144 | 0.0024 | Isopropyl 2 | | |
| **43** | H19 | 0.1049 | 0.1056 | -0.0007 | Isopropyl 2 | | |
| **44** | C12 | -0.2075 | -0.2033 | -0.0042 | Isopropyl 2 | | |
| **45** | H20 | 0.071 | 0.0792 | -0.0082 | Isopropyl 2 | | |
| **46** | H21 | 0.0829 | 0.0777 | 0.0052 | Isopropyl 2 | | |
| **47** | H22 | 0.0768 | 0.0724 | 0.0044 | Isopropyl 2 | | |
| **48** | C13 | -0.2011 | -0.2081 | 0.007 | Isopropyl 2 | | |
| **49** | H23 | 0.0742 | 0.0712 | 0.003 | Isopropyl 2 | | |
| **50** | H24 | 0.0738 | 0.082 | -0.0082 | Isopropyl 2 | | |
| **51** | H25 | 0.0881 | 0.0824 | 0.0057 | Isopropyl 2 | | |
| **52** | C14 | 0.283 | 0.2861 | -0.0031 | Amide | | |
| **53** | C15 | 0.0441 | 0.0469 | -0.0028 | Backbone | | |
| **54** | H26 | 0.1376 | 0.1475 | -0.0099 | Backbone | | |
| **55** | C16 | -0.1357 | -0.1312 | -0.0045 | Backbone | | |
| **56** | H27 | 0.089 | 0.0871 | 0.0019 | Backbone | | |
| **57** | H28 | 0.1027 | 0.089 | 0.0137 | Phenyl 1 | | |
| **58** | C17 | -0.0776 | -0.0766 | -0.001 | Phenyl 1 | | |
| **59** | C18 | -0.1237 | -0.1089 | -0.0148 | Phenyl 1 | | |
| **60** | H29 | 0.128 | 0.1366 | -0.0086 | Phenyl 1 | | |
| **61** | C19 | -0.1267 | -0.1168 | -0.0099 | Phenyl 1 | | |
| **62** | H30 | 0.1301 | 0.1488 | -0.0187 | Phenyl 1 | | |
| **63** | C20 | -0.1265 | -0.1344 | 0.0079 | Phenyl 1 | | |
| **64** | H31 | 0.1264 | 0.1196 | 0.0068 | Phenyl 1 | | |
| **65** | C21 | -0.1223 | -0.1298 | 0.0075 | Phenyl 1 | | |
| **66** | H32 | 0.1294 | 0.1216 | 0.0078 | Phenyl 1 | | |
| **67** | C22 | -0.1193 | -0.1218 | 0.0025 | Phenyl 1 | | |
| **68** | H33 | 0.1283 | 0.1182 | 0.0101 | Phenyl 1 | | |
| **69** | C23 | -0.1968 | -0.1653 | -0.0315 | Backbone | | |
| **70** | H34 | 0.0937 | 0.0762 | 0.0175 | Backbone | | |
| **71** | H35 | 0.0904 | 0.104 | -0.0136 | Backbone | | |
| **72** | C24 | 0.0072 | 0.0102 | -0.003 | Hydroxyl | | |
| **73** | H36 | 0.0996 | 0.0832 | 0.0471 | Hydroxyl | | |
| **74** | C25 | 0.0532 | 0.0094 | 0.0288 | Backbone | | |
| **75** | H37 | 0.1256 | 0.1158 | 0.0184 | Backbone | | |
| **76** | C26 | -0.1443 | -0.1308 | -0.0086 | Backbone | | |
| **77** | H38 | 0.1086 | 0.0914 | 0.0087 | Backbone | | |
| **78** | H39 | 0.1004 | 0.1108 | -0.0102 | Backbone | | |
| **79** | C27 | -0.0753 | -0.0796 | 0.0064 | Phenyl 2 | | |
| **80** | C28 | -0.1234 | -0.1136 | 0.0038 | Phenyl 2 | | |
| **81** | H40 | 0.1314 | 0.1262 | -0.0029 | Phenyl 2 | | |
| **82** | C29 | -0.1295 | -0.1214 | 0.0023 | Phenyl 2 | | |
| **83** | H41 | 0.1298 | 0.1285 | -0.0028 | Phenyl 2 | | |
| **84** | C30 | -0.1284 | -0.1238 | 0.0004 | Phenyl 2 | | |
| **85** | H42 | 0.1302 | 0.1265 | -0.0011 | Phenyl 2 | | |
| **86** | C31 | -0.1246 | -0.1215 | -0.0034 | Phenyl 2 | | |
| **87** | H43 | 0.1305 | 0.1277 | -0.0027 | Phenyl 2 | | |
| **88** | C32 | -0.1201 | -0.1222 | 0.0034 | Phenyl 2 | | |
| **89** | H44 | 0.1279 | 0.1249 | -0.0034 | Phenyl 2 | | |
| **90** | C33 | 0.4051 | 0.4137 | 0.0059 | Carbamate | | |
| **91** | C34 | 0.0768 | 0.1022 | 0.0027 | Backbone | | |
| **92** | H45 | 0.1134 | 0.103 | 0.0023 | Backbone | | |
| **93** | H46 | 0.1034 | 0.09 | 0.0035 | Backbone | | |
| **94** | C35 | -0.4118 | -0.4059 | 0.0026 | Thiazole 2 | | |
| **95** | C36 | -0.126 | -0.0762 | -0.0063 | Thiazole 2 | | |
| **96** | H47 | 0.184 | 0.1828 | -0.0068 | Thiazole 2 | | |
| **97** | C37 | -0.3582 | -0.3094 | -0.0018 | Thiazole 2 | | |
| **98** | H48 | 0.1917 | 0.1782 | -0.0006 | Thiazole 2 | | |

**S4. CSD Torsion Angle Fragment Searching**

A search for similar fragment structures within the CSD consistent with these four torsions revealed that the majority of these had conformations close to planar i.e. 0° or 180° torsion angles (Figure S3.1).


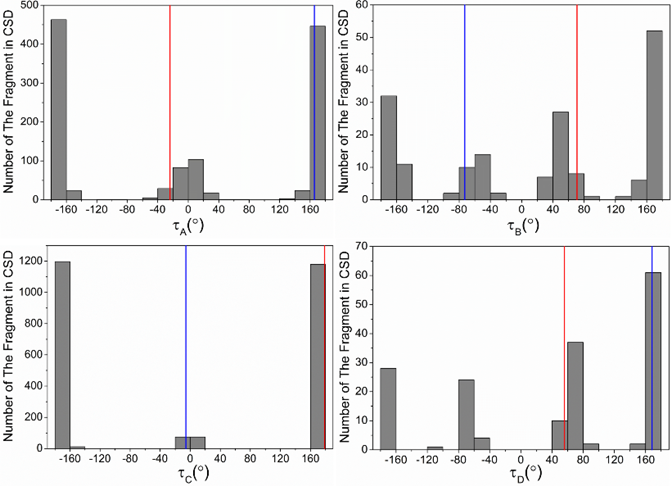


Figure S4.1. Results of the search of the CSD for the constituent fragments which are defined by the 4 key torsions given Table 1 and Figure 5. The red colour refers to form I and blue colour refers to form II.

The torsions for the molecular fragments defined by τ_A_ and τ_D_, for the form II polymorph, were found to be consistent with the majority of crystal structures in the CSD suggesting that form II might be more consistent with a more stable structure when compared to form I. In contrast, τ_C_ of form II is atypical of this type of linkage where the CSD search revealed that this conformation only existed in ca. 5% of the published crystal structures.^33^

**S5. Molecular structures of the important synthons in Forms I and II of ritonavir**


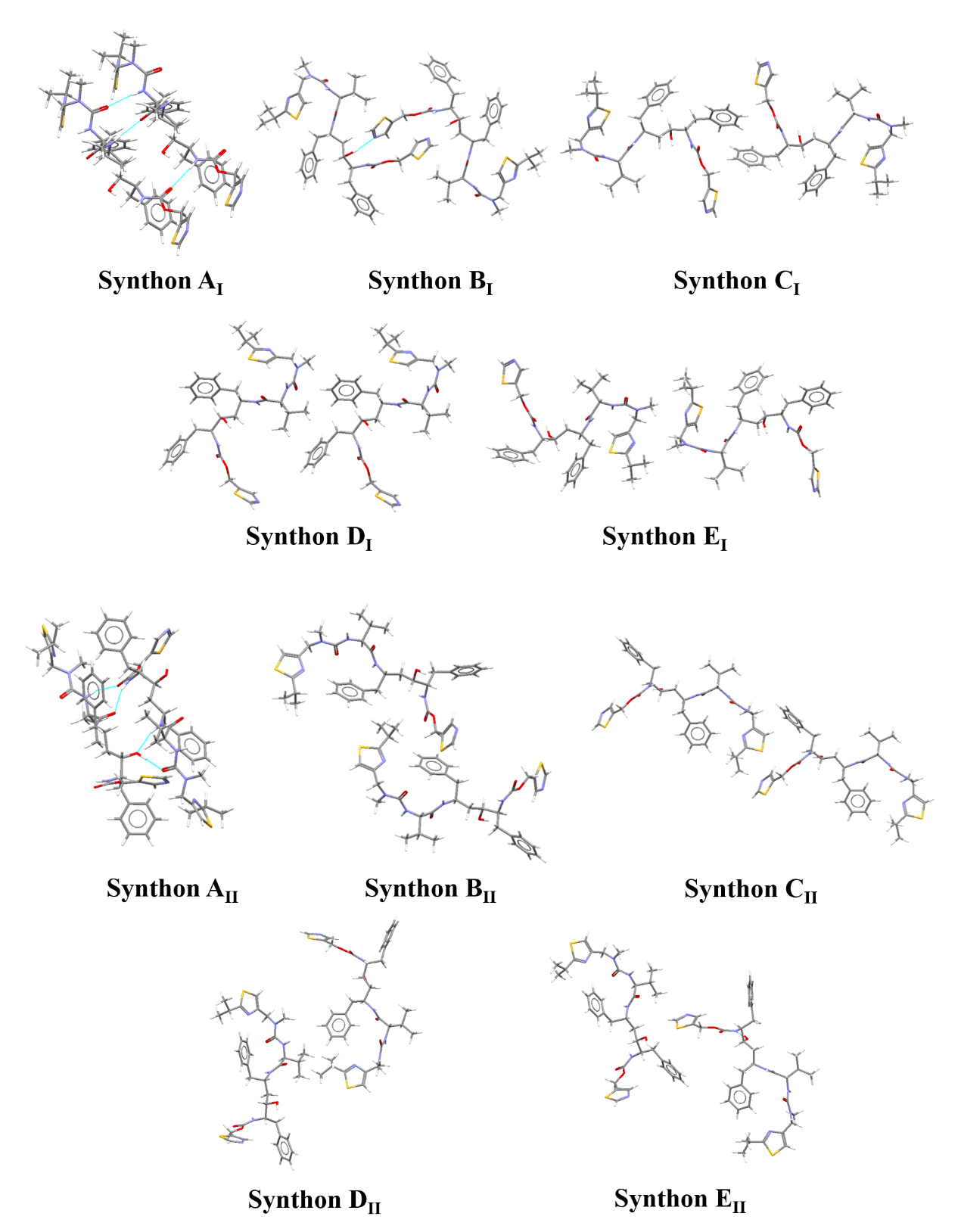


Figure S5.1 Pairwise intermolecular interactions of the five most important synthons (as detailed in Table 6 in the main manuscript) calculated using the atom-atom method based upon the crystal structures of ritonavir form I (top) and form II (bottom).

**S6. Molecular packing diagrams highlighting the different hydrogen bonding motifs in Forms I and II of ritonavir**


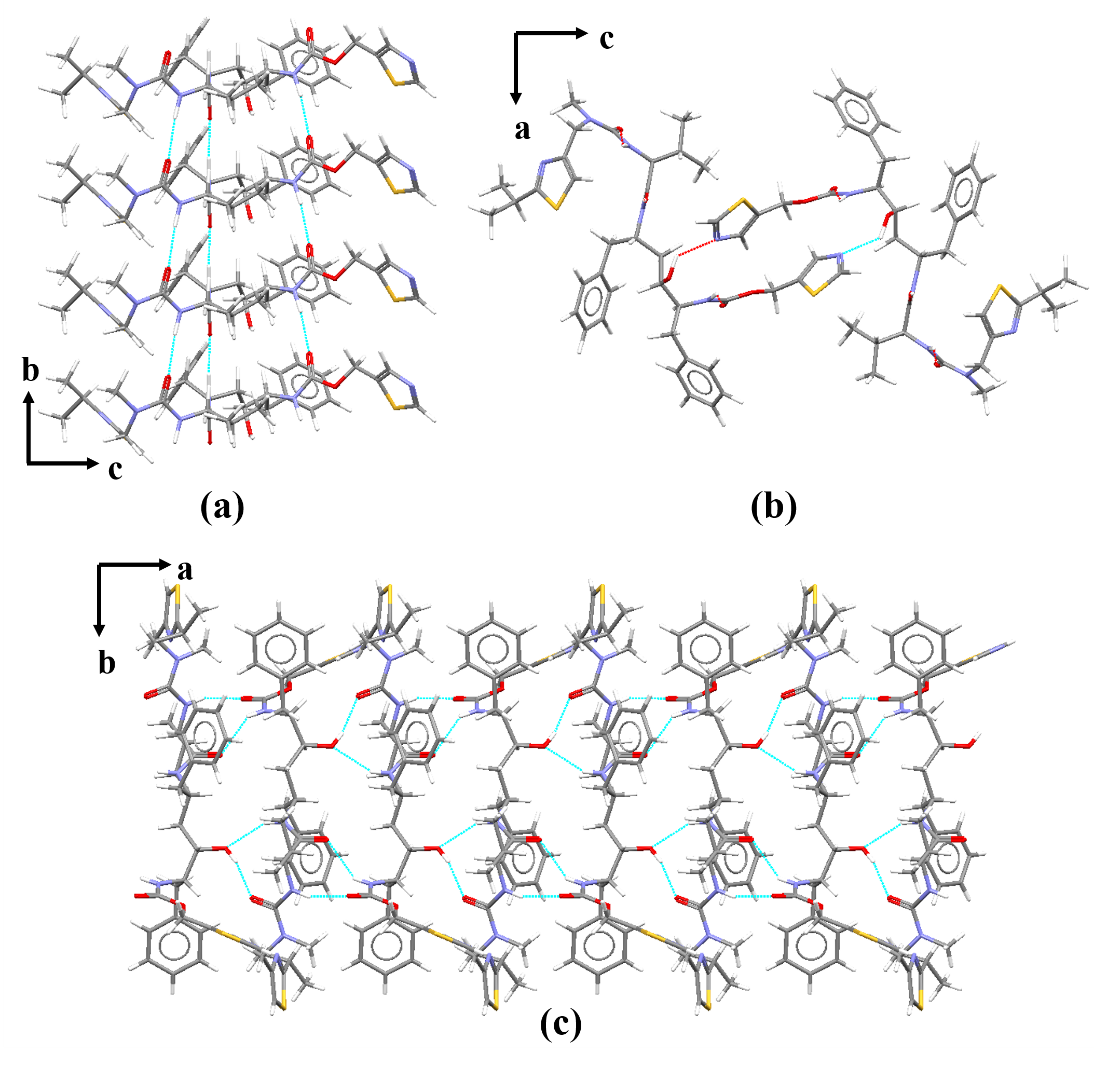


Figure S6.1 Hydrogen bonding mechanisms in the crystal structures of (a) form I along a axis stacks; (b) form I along b axis stacks; and (c) form II along c axis stacks.

**S7. Form I (0 1 1) surface highlighting the H-bonding acceptors exposed at this surface**


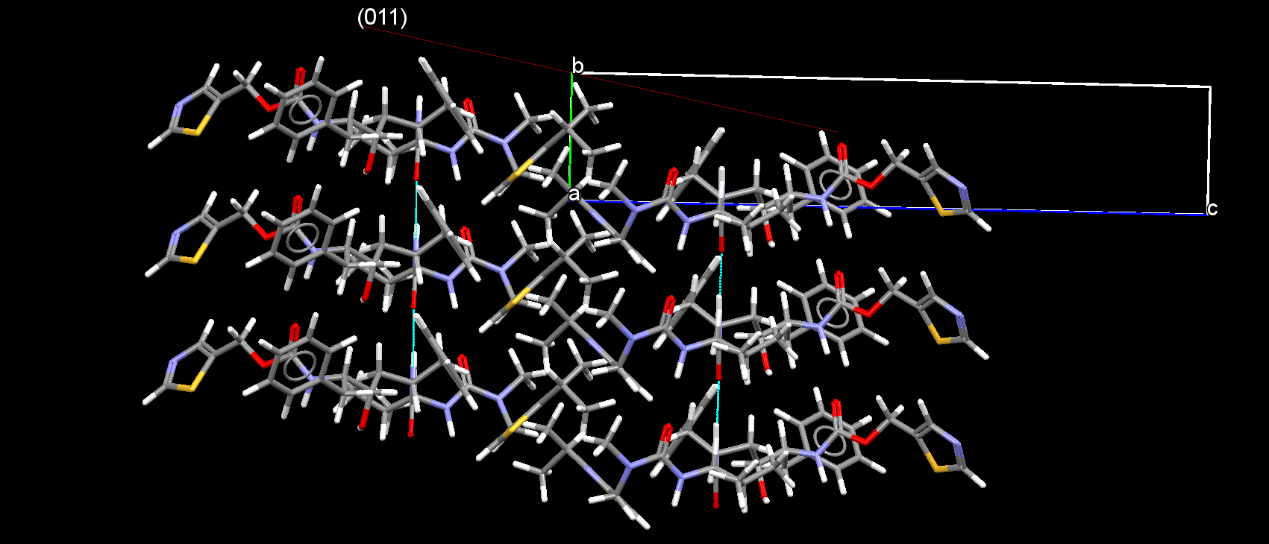


Figure S7. H-bond acceptor exposed on surface (011) of form I.

**S8. Form II hydrogen bonding pattern along the a-axis**


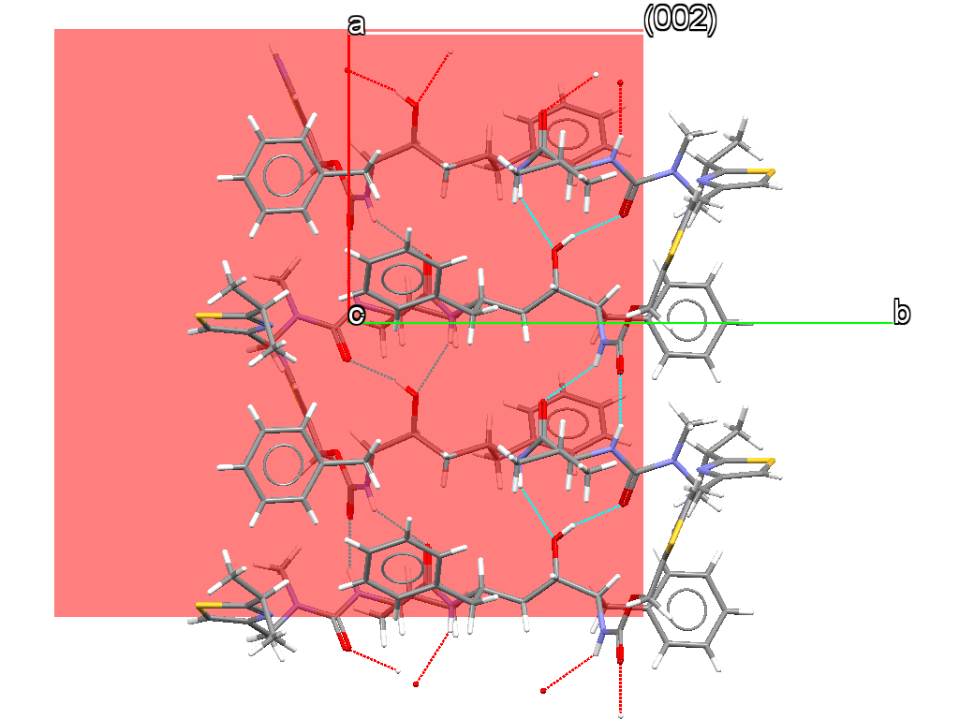


Figure S8.1 The directional hydrogen bonding pattern along the a-axis of form II

#

# S9. Glossary:

vdW: van der Waals

H-bonding: hydrogen bonding

synthons: pairwise intermolecular interactions

d-spacing: interatomic plane separation

intrinsic synthons: fully saturated synthons found in the bulk of the crystal structure

extrinsic synthons: unsaturated synthons due to surface termination of the crystal structure

lattice energy: strength of the intermolecular interactions within the crystal structure per mol

slice energy: energy of intermolecular interactions found within one d-spacing on the (hkl) crystallographic plane

attachment energy: energy of intermolecular interactions formed when a slice one d-spacing thick is added to a surface defined by (hkl) plane
